# Effect of adding Schroth physiotherapeutic scoliosis specific exercises to standard care in adolescents with idiopathic scoliosis on posture assessed using surface topography: A secondary analysis of a Randomized Controlled Trial (RCT)

**DOI:** 10.1371/journal.pone.0302577

**Published:** 2024-04-30

**Authors:** Nada Mohamed, Vivechana Acharya, Sanja Schreiber, Eric C. Parent, Lindsey Westover

**Affiliations:** 1 Department of Mechanical Engineering, University of Alberta, Edmonton, Canada; 2 Department of Civil and Environmental Engineering, University of Alberta, Edmonton, Canada; 3 Department of Physical Therapy, Faculty of Rehabilitation Medicine, University of Alberta, Edmonton, Canada; New York University College of Dentistry, UNITED STATES

## Abstract

**Background:**

Adolescent idiopathic scoliosis (AIS) is a three-dimensional structural asymmetry of the spine and trunk affecting 2–4% of adolescents. Standard treatment is observation, bracing, and surgery for small, moderate, and large curves, respectively. Schroth exercises aim to correct posture and reduce curve progression.

**Purpose:**

This study aimed to determine the effect of Schroth exercises added to the standard care compared to standard care alone on torso asymmetry in AIS.

**Methods:**

In a randomized controlled trial (NCT01610908), 124 participants with AIS (age: 10–18, Cobb: 10°-45°, Risser: ≤3) were randomly assigned to the control (Standard care only) or Schroth (Standard care + Schroth treatment) group. Schroth treatment consisted of 1-hour weekly supervised sessions and 30–45 minutes of daily home exercises for six months. The control group received Schroth exercises in the last six months of the 1-year monitoring period. Markerless 3D surface topography assessed torso asymmetry measured by maximum deviation (MaxDev) and root mean square (RMS). Intention to treat linear mixed effects model analysis was compared to the per protocol analysis.

**Results:**

In the intention to treat analysis, the Schroth group (n = 63) had significantly larger decreased RMS (-1.2 mm, 95%CI [-1.5,-0.9]mm, p = 0.012) and MaxDev (-1.9mm, 95%CI [-2.4,-1.5]mm, p = 0.025) measurements compared to controls (n = 57) after six months of intervention. In the per protocol analysis (Schroth n = 39, control n = 36), the Schroth group also had a significantly larger decrease compared to the control in both the RMS (-1.0mm, 95%CI [-1.9, -0.2]mm, p = 0.013) and MaxDev measurements (-2.0mm, 95%CI [-3.3,-0.5]mm, p = 0.037). For the control group, both the intention to treat and per protocol analysis showed no difference in RMS and MaxDev in the last six months of Schroth intervention (p>0.5).

**Conclusion:**

Schroth Exercise treatment added to standard care (observation or bracing) reduced asymmetry measurements in AIS. As expected, a greater effect was observed for participants who followed the prescribed exercise treatment per protocol.

## Introduction

Adolescent idiopathic scoliosis (AIS) is paediatric condition, that leads to structural and morphological changes of the spine and trunk in all three planes of the body [1]. During adolescent growth, curves progress quickly, and frequent follow-ups are needed to monitor progression [2]. The Scoliosis Research Society (SRS) outlines treatment options which include observation (using frontal plane radiographs taken at regular intervals during growth) for patients with AIS having curves with Cobb angle <25°, bracing for curves 25°- 45°, and elective surgery for growing children with curves >45°[3]. In addition to those modalities, the Society on Scoliosis Orthopedic Rehabilitation Treatment (SOSORT) guidelines recommends physiotherapeutic scoliosis specific exercises (PSSE) for smaller curves and as an add-on to bracing [4].

The Schroth method is a PSSE approach aimed at recalibrating the postural alignment, capitalizing on the motor learning and control. It includes segmental realignment of the trunk, pelvis and legs, using specific eccentric and isometric muscle tension, and corrective breathing exercises to recalibrate the normal postural alignment [5]. Studies on AIS have shown positive results of Schroth exercises on Cobb angle, pain, self-image, vital capacity, and muscle endurance [6–8].

SOSORT Guidelines rank aesthetics as the most important goal when treating patients with AIS, which can be achieved through exercises [4, 9, 10]; however, very few studies on scoliosis address objective measurements of posture [10]. This is partly due to limited methods for measuring aesthetics and posture outcomes [10]. Most systems used clinically to measure aesthetics are based on photograph comparison between visits, with the implicit limitation that photographs are a 2D representation of a 3D condition [11]. Aesthetics can be quantified using surface asymmetry tools such as trunk asymmetry scales or surface topography (ST) [4].

In addition, the effect of Schroth exercises on external asymmetries of the trunk has not been adequately studied [12]. One study reported waist asymmetry during a 24-week Schroth therapy program [13]. Cosmetic changes were also investigated to monitor the effects of other PSSE programs. The lateral deviation of the trunk after an intense rehabilitation program was evaluated in a pilot study by *Schumann et al* [14]. *Weiss et al* used surface topography to monitor the effect Activities of Daily Living (ADL) approach [15]. The scarcity of studies reporting quantitative measurement of external trunk asymmetries during conservative treatment in addition to scoliosis-specific exercises highlights the need for studies to investigate the efficacy of PSSE on cosmetic outcomes. To address this critical gap, asymmetry measurement parameters from markerless surface topography analysis (ST) may be used to monitor posture outcomes during treatment. The proposed ST measurement parameters, which are developed using the whole torso surface information, have not previously been monitored during prescribed PSSE treatment programs.

Asymmetry analysis using ST can identify severity and monitor the effect of progression of scoliosis on torso posture [16–20]. ST can quantify external torso asymmetry associated with scoliosis, and reduce the use of harmful radiographs for mild curves and those without curve progression [19, 21]. A 3D markerless asymmetry analysis ST technique has been developed that assesses the 3D geometry of the torso by using the best sagittal plane of symmetry [16, 22, 23]. This technique is reliable in identifying the location and direction of scoliosis curves by analyzing the severity of asymmetry [17]. Extracted measurements from ST and decision trees help identify curve severity at a single visit and detect whether curves have progressed (>5° increase in Cobb angle) using scans from two temporally separated visits [18, 20].

In this randomized controlled clinical trial (RCT), the objective was to determine the effect of the 6-month Schroth exercise intervention added to standard care compared to standard care used alone on asymmetry measures in AIS using ST analysis. Additionally, we determined the impact of offering Schroth exercises to participants from the control group during the last half of the 1-year follow-up. We hypothesized that the Schroth exercise program added to standard care would be superior in reducing asymmetry compared to standard care alone.

## Methods

### Ethics statement

This study has been approved by The University of Alberta Health Research Ethics Board (Pro00043397) and includes data collected from a pilot study (Pro00011552). The study reports a secondary analysis of data from a registered randomized controlled trial (NCT01610908). Its primary objective was to ascertain the impact of a six-month Schroth PSSE intervention added to standard care versus standard care alone on the Cobb angle in participants with AIS [7]. The CONSORT checklist is available in S1 File. Participants provided signed assent and parents provided signed parental informed consent.

**Fig 1:**
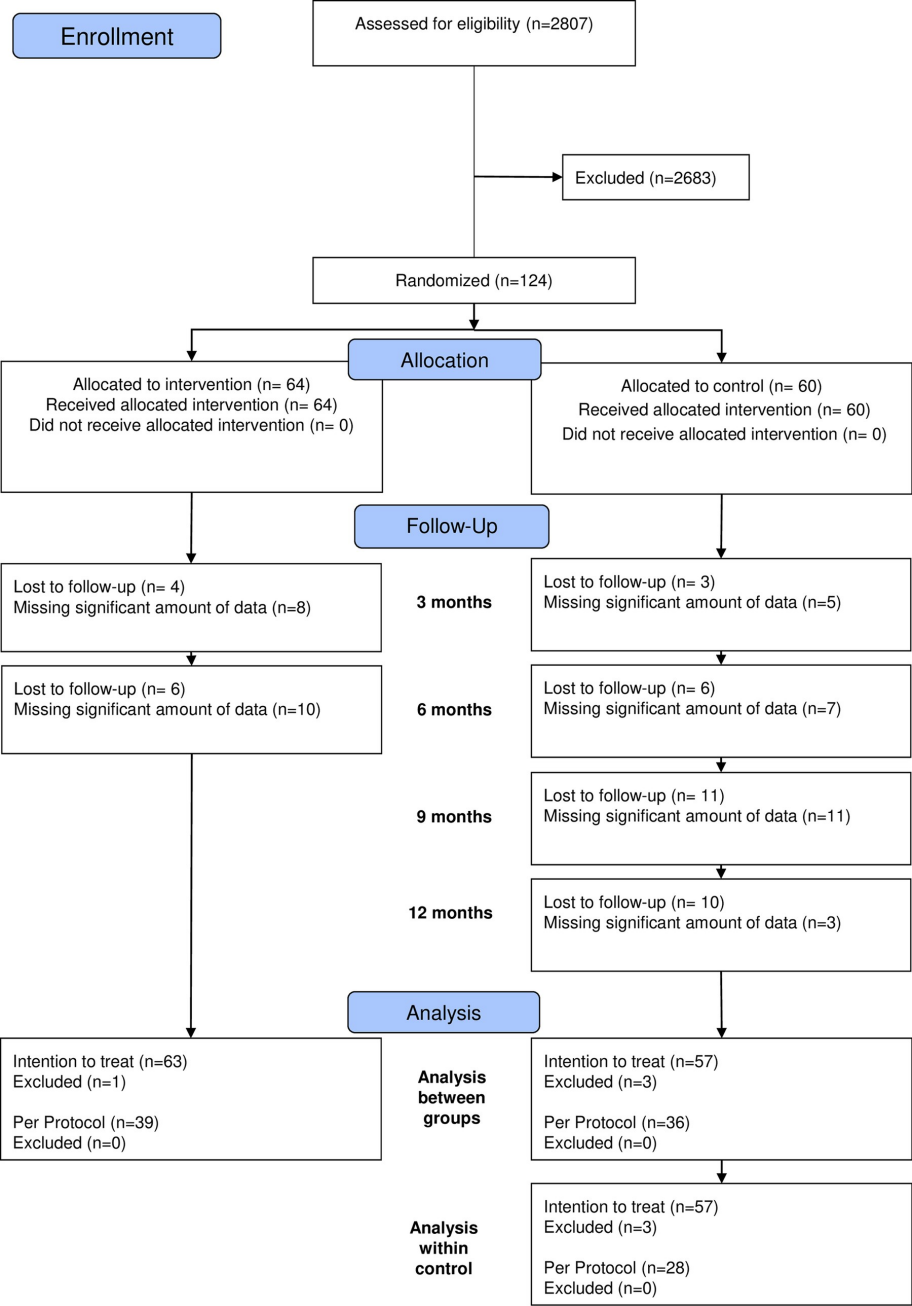
CONSORT flow-chart showing intention to treat and per protocol group sample sizes.

A total of 124 patients with AIS were recruited from the Edmonton Scoliosis Clinic. Participants were between 10 and 18 years old with curves between 10° and 45° [7]. Participants were enrolled from April 2011 to December 2019. Recruitment of the first 50 participants during the pilot study included both sexes and Risser grades 0–5. The remaining 74 participants were restricted to only females with Risser grade ≤3. The selection criteria were modified following the pilot study to target patients with higher risk of progression. Recruitment of participants ceased when funding of the trial ended. The CONSORT flow chart is presented in Fig 1.

In a parallel design, participants were randomly allocated to two groups: Control (n = 60) or Schroth (n = 64). The randomization sequence was stratified for four Schroth curve types and used random block sizes. Randomization was performed by the research coordinator using REDCAP to ensure the concealment of the randomization sequence [24].

The Schroth group was offered a 6-month supervised Schroth physiotherapeutic scoliosis specific exercises (PSSE) intervention combined with standard care. The treatment consisted of five one-hour private sessions during the first two weeks, followed by weekly one-hour private classes for six months and a 30–45 min daily home exercise program. After completing the 6-month supervised intervention, participants were advised to continue with the program, but the supervision was discontinued. An algorithm for determining scoliosis curve types and prescribing Schroth exercise based on curve type was used [25, 26]. Exercise treatments were offered by four certified Schroth therapists over the trial duration aiming to pair individual participants with the same therapist.

Adherence was monitored using logbooks and session attendance. To maximize adherence, equipment and exercise description handouts were provided. Participants were asked to discuss their home exercise environment with their therapist. Parents were also asked to sign logbooks weekly, and adherence was discussed at every visit.

The control group received only standard care for the first six months, including observation or rigid bracing prescribed based on SRS recommendations [27]. During the last six months of the 1-year follow-up period, the control group underwent Schroth therapy. The complete study protocol has been made available in S2 File and published [24].

### Asymmetry analysis

The ST analysis method was previously reported in the literature [16–20]. Entire torso ST scans were collected at baseline, three, six, and 12 months by an examiner blinded to group allocation. Following the completion of data collection, all gathered information was anonymized to ensure the protection of participants’ identities.

**Fig 2:**
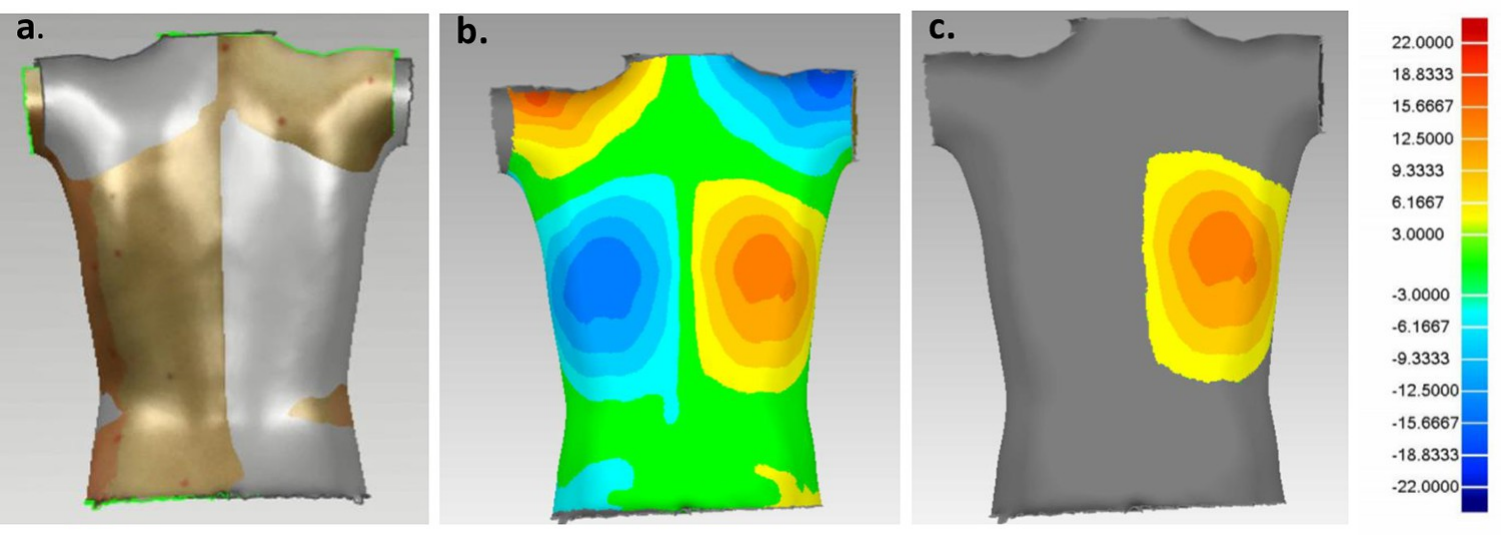
Surface topography procedure. (a) Alignment of the reflected (gold) and original torso (grey), (b) deviation color map where red reflects area of protrusion and blue to areas of depression relative to the other side, and (c) isolated patch of interest to calculate measurement parameters.

The four views, including both sides, front and back from the ST cameras were imported into Geomagic Control 2015 (3D Systems, North Carolina, USA) for merging and cropping to retain a full 3D torso. For the asymmetry analysis, the torso model was duplicated and reflected about the midsagittal plane. The reflected torso was then aligned with the original (Fig 2A) to minimize the distance between the models. The roto-inversion plane associated with this transformation was termed the plane of best symmetry [22, 23]. A deviation color map (DCM) was created, illustrating the magnitude of the distances between each point on the original torso and its corresponding point on the reflected torso (Fig 2B). The threshold to separate normal surface variations from relevant asymmetry was ±3mm [16]. However, in cases with maximum deviations greater than 9.33mm, a threshold value of ±9.33mm was also applied to ensure separation between asymmetry patches [19]. Color patches illustrating the asymmetry were isolated by removing points with deviations below the defined threshold values (Fig 2C) using a custom algorithm (Wolfram Mathematica v. 12.1, Wolfram Research Inc., Illinois, USA) [16].

For each isolated asymmetry patch, the maximum deviation (MaxDev) and root mean square (RMS) of the deviations were determined [16]. The RMS and MaxDev parameters for the largest patch by area were recorded for each patient at each time point. Patches on the shoulder were excluded since they do not show a corresponding curve on the radiograph and can manifest through uneven positioning of the shoulders [17]. Patches around the hip were also excluded as they might be affected by model cropping. ST analysis was completed by evaluators blinded to group allocation. The trunk asymmetry parameters of RMS and MaxDev are related to the cosmetic score stated in the protocol (S2 File). The cosmetic score quantifies the visual appearance of trunk asymmetry, which is directly captured by the RMS value indicating the overall magnitude of asymmetry and the MaxDev value representing the most prominent deviation.

### Longitudinal asymmetry evaluation

**Fig 3:**
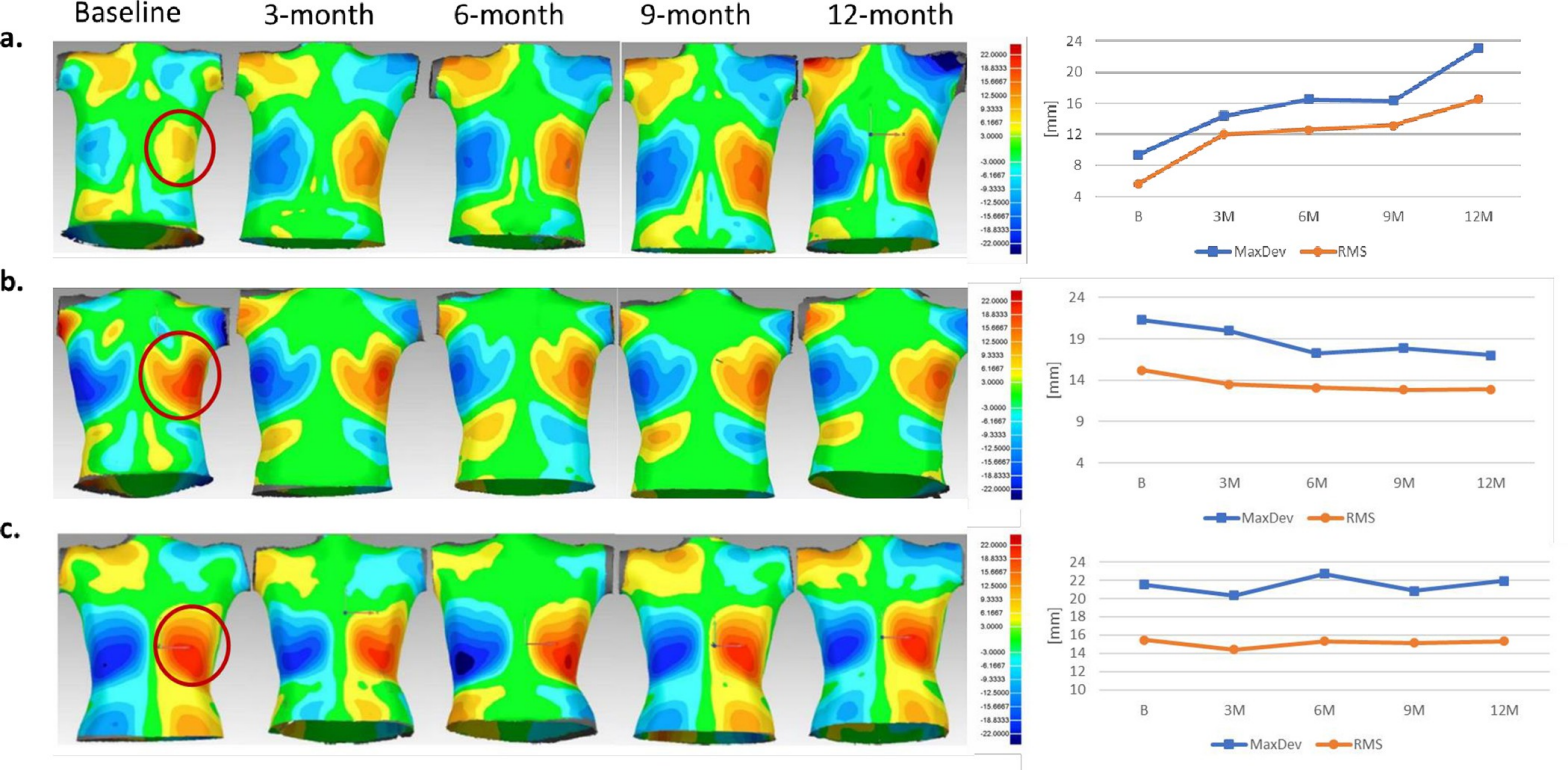
Examples of deviation colour maps, RMS and MaxDev measurements of the largest patch by area over time. (a) Patient experiencing progression, (b) patient with asymmetry improvement, and (c) no change in asymmetry.

Participants were classified as experiencing progression, improvement, or no change during the first six months. A previously published classification tree was used to classify curves as either progression (increase in Cobb of ≥5°) or non-progression [20]. To classify improvement, measurements were plotted within participants over time to evaluate trendlines. A patient with asymmetry worsening showed an increasing trendline and increasing intensity of their color patch (Fig 3A). In contrast, the opposite is shown for a patient with asymmetry improvement (Fig 3B). A constant trendline is observed for a patient without changes in asymmetry (Fig 3C). Using a sub-sample of 50 participants (25 Schroth and 25 control) and by visual inspection, a threshold of -2.2mm for ΔRMS and ΔMaxDev was determined for asymmetry improvement classification. To avoid bias, the classification was applied blinded to group allocation.

### Statistical analysis

A covariate-adjusted linear mixed effects model was used to identify significant differences in RMS and MaxDev between Schroth and control groups. The initial analysis reported the significance of the group, time, and interaction effects. Final analyses were reported after dropping the effects that were not significant or dropping the main effects of time and group when interactions were significant. Separate linear mixed effects model analyses were carried out to compare the control group data from their last six months of the trial during which they had received Schroth exercises to the initial six months while they had not received exercises. Covariates included in the model were age, weight, height, scoliosis curve classification, and whether participants were wearing a brace. The covariance structure for each outcome were selected based on minimizing the Akaike information (AIC) value.

The proportions of participants classified as demonstrating progression, improvement, or no change in their asymmetry were compared between Schroth and Control groups using a Pearson Chi-square test. The significance level was set at p≤0.05.

Intention to treat and per protocol analyses were performed. The intention to treat analysis considered the entire sample, including dropouts and those with missing data. The per protocol analysis only included participants who completed the study per the prescribed protocol. To keep participants who dropped out or with missing data in the analysis, multiple imputation was used. The imputation model included all variables in the analysis model, and predictive mean matching (PMM) was used to impute the missing values [28]. Five complete datasets were obtained and analysed separately, and the resulting parameter estimates were then pooled across the imputed data sets using Rubin’s rules [29]. Finally, Post hoc tests using Sidak adjustments to control for Type I error were conducted. In the intention to treat analysis, we opted for the implementation of multiple imputation as opposed to the last value carried forward technique stated in the protocol (S2 File). The carry forward method may introduce bias by assuming that missing values remain constant over time [28]. The SAS software was used for statistical analyses [30]. We report significant main effects to show the influence of individual independent variables, as well as significant interactions to show specific relationships among multiple variables and their collective impact on the outcome.

## Results

Thirty-two of the 124 participants were lost to follow up or no longer wished to participate in the study. Seventeen of the 124 participants had scans that were of poor quality, or their 3D torso reconstruction was impossible because of missing data. Table 1 reports baseline characteristics of intention to treat and per protocol analyses between groups. The study data are available in S4 File.

**Table 1:** Patient characteristics at baseline for the Schroth and control groups for the intention to treat and per protocol analyses.

	Intention to Treat		Per Protocol	
	Control	Schroth	Control	Schroth
	N = 57	N = 63	N = 36	N = 39
**Age (yrs)**	13.0 ± 1.7^a^	13.1 ± 1.8^a^	13.2 ± 1.81.8^a^	13.3 ± 1.8^a^
**Height (cm)**	156.4 ± 9.0^a^	155.6 ± 11.0^a^	156.9 ± 9.8^a^	156.0 ± 10.4^a^
**Weight (kg)**	46.6 ± 9.4^a^	44.1 ± 10.7^a^	46.9 ± 9.1^a^	43.3 ± 9.3^a^
**Braced (%)**	40 (70)^b^	41 (65)^b^	26 (72)^b^	24 (62)^b^
**Number of girls (%)**	56 (98)^b^	58 (92)^b^	36 (100)^b^	36 (92)^b^
**Maximum Cobb Angle (°)**	27.7 ± 9.1^a^	26.5 ± 8.2^a^	29.5 ± 8.3^a^	26.3 ± 8.5^a^
**Risser**	R0(26;46%)^b^	R0(31;49%)^b^	R0(16;44%)^b^	R0(20;51%)^b^
	R1(4;7%)^b^	R1(2;3%)^b^	R1(3;8%)^b^	R1(1;3%)^b^
	R2(7;12%)^b^	R2(5;8%)^b^	R2(5;14%)^b^	R2(1;3%)^b^
	R3(2;4%)^b^	R3(8;13%)^b^	R3(0;0%)^b^	R3(7;18%)^b^
	R4(4;7%)^b^	R4(3;5%)^b^	R4(2;6%)^b^	R4(3;8%)^b^
	R5(9;16%)^b^	R5(5;8%)^b^	R5(7;19%)^b^	R5(2;5%)^b^
**Curve Type**	3c(5;9%)^b^	3c(9;14%)^b^	3c(3;8%)^b^	3c (5;13%)^b^
	3cp(20;35%)^b^	3cp(18;29%)^b^	3cp(13;36%)^b^	3cp (11;28%)^b^
	4c(7;12%)^b^	4c(8;13%)^b^	4c(4;11%)^b^	4c (6;15%)^b^
	4cp(25;44%)^b^	4cp(28;44%)^b^	4cp(16;44%)^b^	4cp (17;44%)^b^
**RMS (mm)**	11.1 ± 4.1^a^	12.2 ± 4.6^a^	11.1 ± 4.0^a^	12.3 ± 5.2^a^
**MaxDev (mm)**	15.2 ± 6.4^a^	17.4 ± 8.0^a^	15.4 ± 6.2^a^	18.0 ± 8.9^a^

^a^ Mean ± standard deviation

^b^ Frequency N (percent of whole sample)

### Intention to treat comparisons of changes in RMS and MaxDev

**Fig 4:**
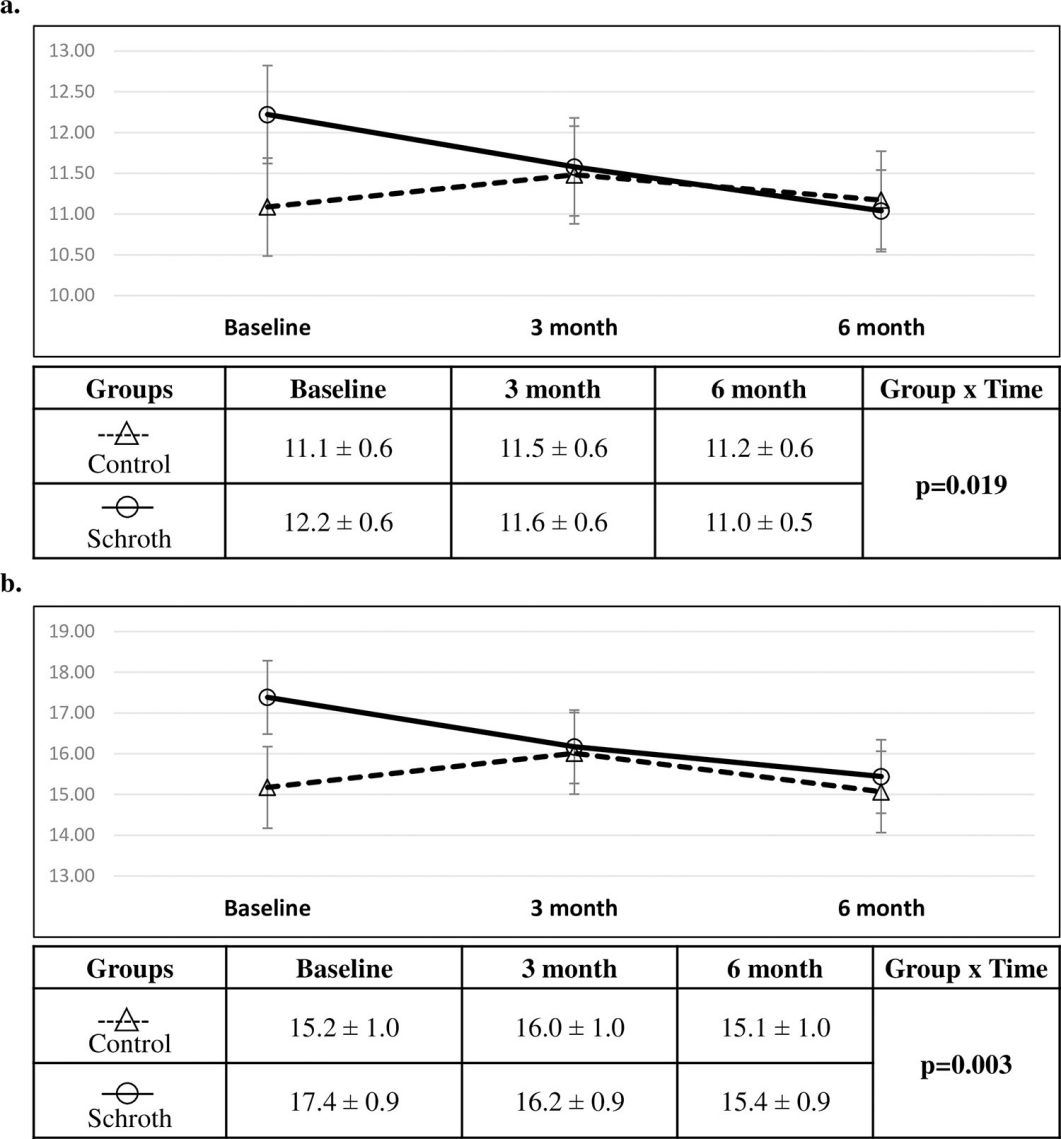
Comparison between groups from intention to treat analysis (mean ± SE). Comparison of (a) RMS (mm) and (b) MaxDev (mm) outcomes. Linear mixed effects analysis produced the p-value presented.

For the RMS outcome, group and time showed non-significant main effects (group effect p = 0.651, time effect p = 0.681), while their interaction was significant (time-by-group interaction p = 0.03). The interaction was also significant in the reduced model without the main effects of group and time (time-by-group interaction p = 0.019). (Fig 4). Only age covariate was significant in the reduced model (p = 0001). For the MaxDev outcome, group and time showed non-significant main effects (group effect p = 0.157, time effect p = 0.896), while their interaction was significant (time-by-group interaction p = 0.040). The interaction was also significant in the reduced final model (time-by-group interaction p = 0.003). (Fig 4). Only age covariate was significant in the reduced model (p = 0002). AIC was found to be the lowest using the Variance Components covariance structure in the linear mixed models for RMS and MaxDev outcomes. The final linear mixed effects model coefficient estimates are tabulated for RMS and MaxDev outcomes in S3 File. From baseline to six months, post hoc comparison of the estimated marginal means revealed significant ST posture measurements of RMS and MaxDev improvement in the Schroth group from baseline to six months (p = 0.001 and p = 0.0002, respectively). No other significant improvement or worsening was observed in the control group. The complete post hoc analysis results are tabulated in S3 File.

The Schroth group had a decrease in RMS by 4.9% (-0.6mm, 95%CI [-0.9,-0.4]mm) at three months, and 9.8% (-1.2 mm, 95%CI [-1.5,-0.9]mm) at six months compared to baseline. In the control group, RMS increased by 3.6% (0.4mm, 95%CI [0.08, 0.7]mm) at three months and increased by 0.9% (0.08mm, 95%CI [-0.3, 0.5]mm) at six months compared to baseline. Additionally, the Schroth group had a decrease in MaxDev by 6.9% (-1.2mm, 95%CI [-1.7,-0.7]mm) at three months, and 11.5% (-1.9mm, 95%CI [-2.4,-1.5]mm) at six months compared to baseline. In the control group, MaxDev increased by 5.3% (0.8mm, 95%CI [0.4, 1.3]mm) at three months and decreased by 0.7% (-0.1mm, 95%CI [-0.6, 0.4]mm) at six months compared to baseline (Fig 4).

### Per protocol comparisons of changes in RMS and MaxDev

**Fig 5:**
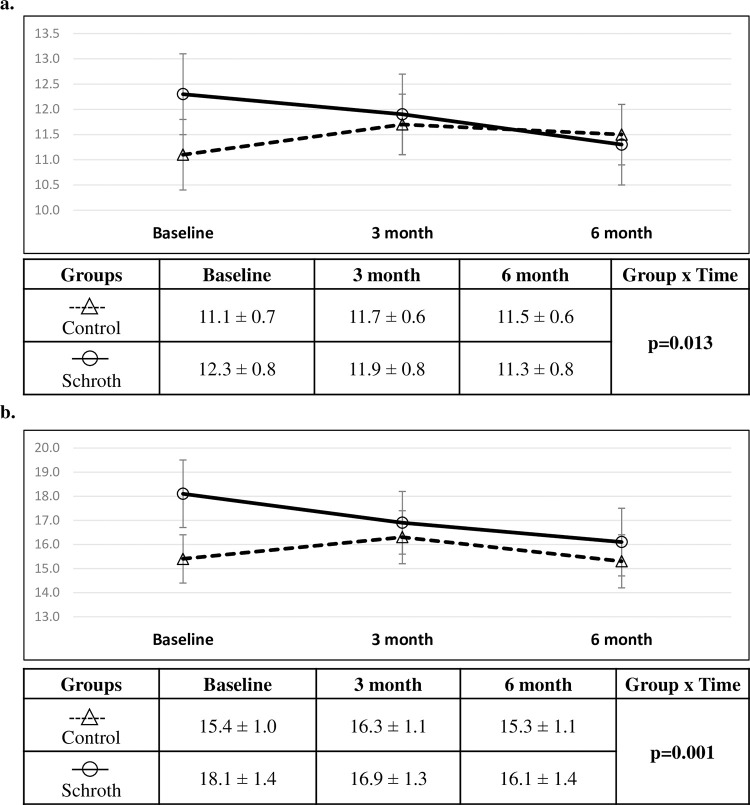
Comparison between groups from the per protocol analysis (mean ± SE). Comparison of (a) RMS (mm) and (b) MaxDev (mm) outcomes. Linear mixed effects analysis produced the p-value presented.

For the RMS outcome, group showed non-significant main effect (group effect p = 0.452), while time and time-by-group interaction were significant (time effect p = 0.028, time-by-group interaction p = 0.041). The interaction was also significant in the reduced model without the main effects of group and time (time-by-group interaction p = 0.013). Only age and curve classification covariates were significant (p = 0001, p = 0.022, respectively). (Fig 5). For the MaxDev outcome, group showed non-significant main effect (group effect p = 0.248), while time effect and time-by-group interaction were significant (time effect p = 0.002, time-by-group interaction p = 0.050). The interaction was also significant in the reduced final model (time-by-group interaction p = 0.001). (Fig 5). Only age and curve classification covariates were significant (p = 0002, p = 0.040, respectively. The final linear mixed effects model coefficient estimates are tabulated for RMS and MaxDev outcomes in S3 File. From Baseline to six months, post hoc comparison revealed significant ST posture measurements of RMS and MaxDev improvement in the Schroth group from baseline to six months (p = 0.01 and p = 0.001, respectively). No other significant improvement or worsening was observed in the control group. The complete post hoc analysis results are tabulated in S3 File.

Over the six months follow-up, the Schroth group had a decrease in RMS by 3.2% (-0.4mm, 95%CI [-1.3,0.4]mm) at three months, and 8.1% (-1.0mm, 95%CI [-1.9, -0.2]mm) at six months compared to baseline. In the control group, RMS increased by 5.4% (0.6mm, 95%CI [-0.3, 1.5]mm) at three months and increased by 3.6% (0.4mm, 95%CI [-0.7, 1.5]mm) at six months compared to baseline. Additionally, the Schroth group had a decrease in MaxDev by 6.6% (-1.2 mm, 95%CI [-2.6,0.3]mm) at three months, and 11.0% (-2.0mm, 95%CI [-3.3,-0.5]mm) at six months compared to baseline. In the control group, MaxDev increased by 5.8% (0.9 mm, 95%CI [-0.6,2.4]mm) at three months and decreased by 0.6% (-0.1mm, 95%CI[-1.7,1.5]mm) at six months compared to baseline (Fig 5).

### Intention to treat group comparison based on the classification

Proportions of patients classified as presenting asymmetry improvement were 32% in the Schroth group and 18% in the control group after six months (Table 2). Participants whose asymmetry progressed were 35% and 19% in the control and Schroth group, respectively. Both groups had similar percentages with no change in asymmetry measurement (Schroth = 49%; control = 47%). Differences in distribution among these categories between groups did not reach significance (Chi-square = 2.396, *p* = 0.092; Table 2). Combining improved and no change as a positive outcome, 81% of Schroth participants had a positive outcome compared to 65% of controls. This difference also did not reach statistical significance (Chi-square = 2.278, *p* = 0.137; Table 2).

**Table 2:** Proportions of participants reported as N (percent of group sample) showing improvement, no change or progression in asymmetry after six months from the intention to treat and per protocol analyses.

Group	Asymmetry Improvement N (%)	No change N (%)	Progression N (%)	Pearson hi-square 3 categories	Pearson Chi-square Improve or no change vs progression
**Intention to Treat**					
Control	10 (18)	27 (47)	20 (35)	p = 0.092	p = 0.137
Schroth	20 (32)	31 (49)	12 (19)		
**Per Protocol**					
Control	5 (14)	16 (44)	15 (42)	p = 0.072	p = 0.083
Schroth	12 (31)	19 (49)	8 (21)		

### Per protocol group comparison based on the classification

In the per protocol analysis, the percentage of participants classified as presenting with improved asymmetry was 31% in the Schroth group and 14% in the control group (Table 2). The control group had 42% of their participants whose asymmetry worsened. In contrast, 21% of the participants in the Schroth group exhibited asymmetry progression. Stable asymmetry measurements were 49% in the Schroth group and 44% in the control group. Differences between groups in distribution among the three categories did not reach statistical significance (Chi-square = 5.158, *p* = 0.072; Table 2). The percentage of participants classified as presenting positive outcomes was 79% in the Schroth group and 63% in the control group, but differences did not reach statistical significance (Chi-square = 3.008, p = 0.083; Table 2).

### Intention to treat analysis of control group when receiving delayed Schroth exercises

**Fig 6:**
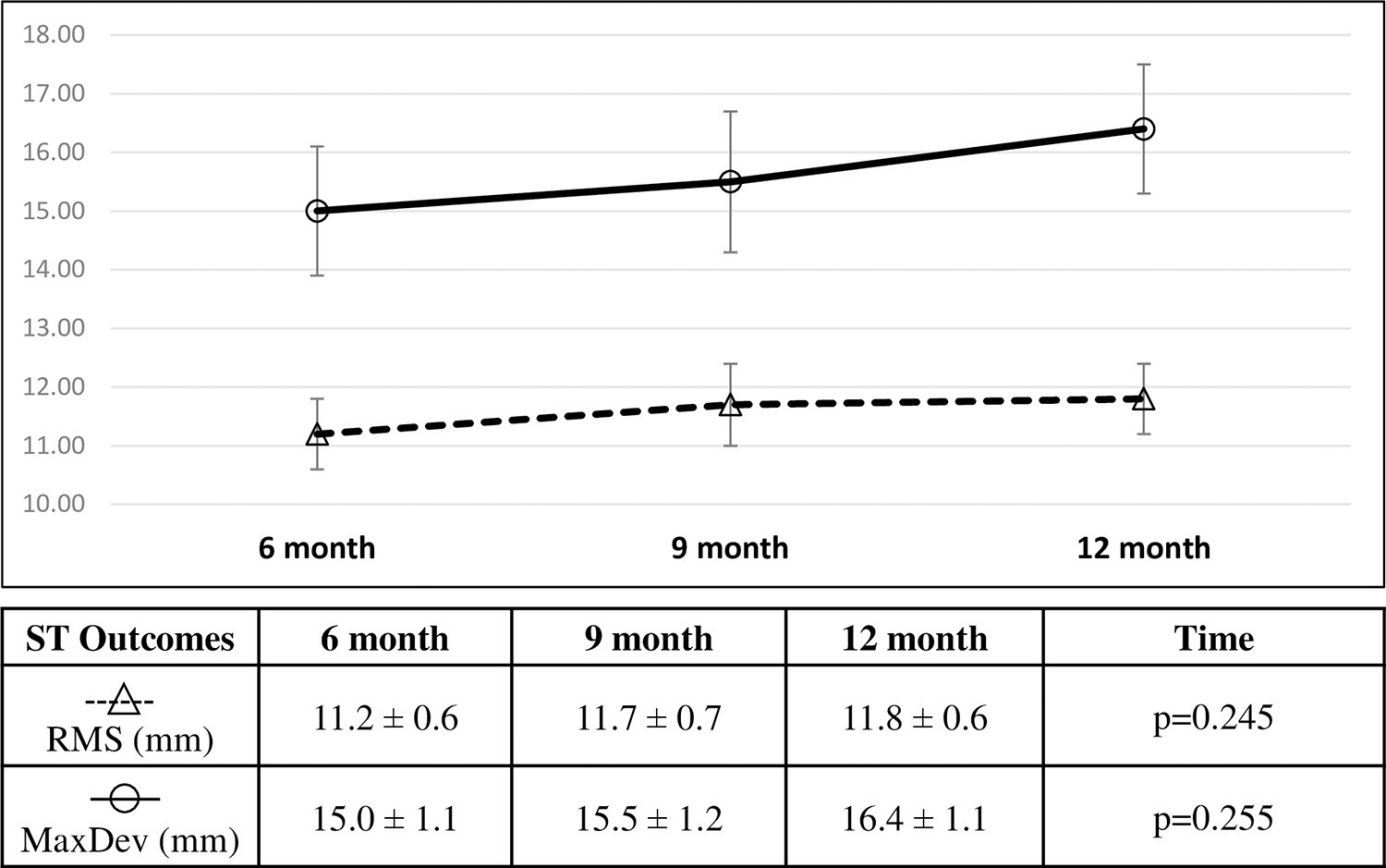
Average RMS (mm) and MaxDev (mm) in the group serving as control during the first 6 months and receiving exercises in the last 6 months presented over time from the intention to treat analysis (mean ± SE). Linear mixed effects analysis produced the p-value presented.

The time factor in the mixed model for the delayed Schroth exercises in the control group was not significant for the RMS measurements from nine months to 12 months (p = 0.245). Likewise, difference in MaxDev over time was not significant from nine months to 12 months (p = 0.255). Percent change of RMS was 4.5% (0.5mm, 95% CI [0.1,0.9]mm) from six to nine months, and 5.4% (0.6mm, 95% CI [0.2,1.0]mm) from six to 12 months (Fig 6). Percent change of MaxDev was 3.3% (0.5mm, 95% CI [-0.04,1.0]mm) from six to nine months, and 9.3% (1.3mm, 95% CI [0.8,1.9]mm) from six to 12 months.

### Per protocol analysis of control group when receiving delayed Schroth exercises

**Fig 7:**
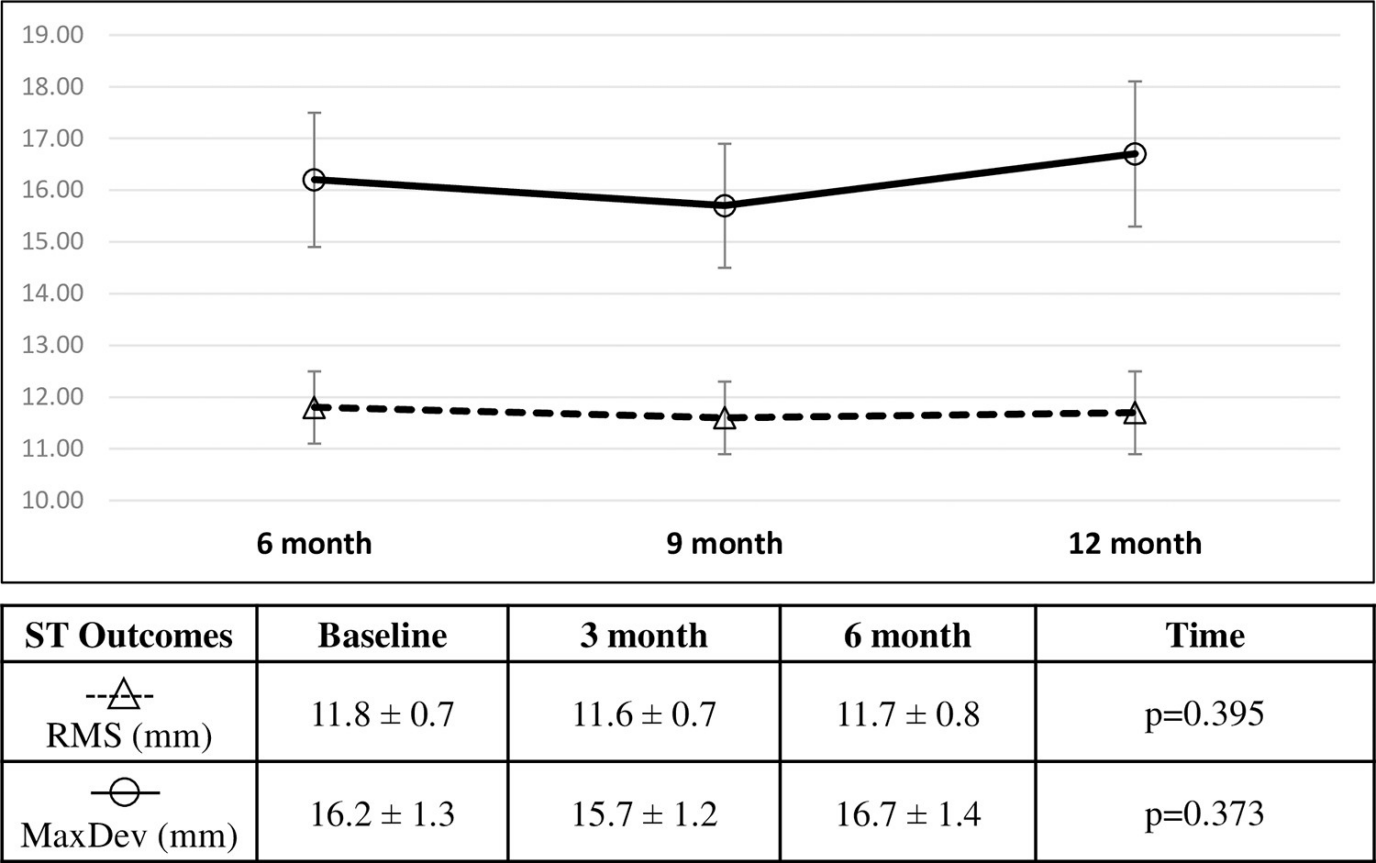
Average RMS (mm) and MaxDev (mm) in the group serving as control during the first 6 months and receiving exercises in the last 6 months presented over time from the per protocol analysis (mean ± SE). Linear mixed effects analysis produced the p-value presented.

The time factor in the linear mixed effects model for the delayed Schroth exercises in the control group was not significant for the RMS measurements from nine months to 12 months (p = 0.395). Likewise, difference in MaxDev over time was not significant from nine months to 12 months (p = 0.373,). Percent change of RMS was -1.7% (-0.2mm, 95% CI [-1.3,1.0]mm) from six to nine months, and -0.8% (-0.1mm, 95% CI [-1.3,1.2]mm) from six to 12 months. Percent change of MaxDev was -3.1% (-0.5mm, 95% CI [-2.1,1.0]mm) from six to nine months, and 3.1% (0.5mm, 95% CI [-1.1,2.1]mm) from six to 12 months (Fig 7).

## Discussion

For both RMS and MaxDev measurements, a significant interaction effect between group and time was determined in the intention to treat and per protocol analyses, suggesting that the longitudinal changes depend on the group (Schroth or control). During the six months intervention period, RMS and MaxDev significantly decreased in the Schroth group. In addition, MaxDev had a greater reduction compared to RMS measurement. Both the intention to treat and per protocol analyses supported the same finding. The linear mixed model analyses revealed age and curve classification had significant effect on the outcomes. Age had a significant main effect for both MaxDev and RMS, such that older participants had higher RMS and MaxDev. Patients classified as 4C curve types had better RMS and MaxDev compared to those classified as 3C. Similarly, patients classified as 4CP, had better RMS and MaxDev compared to those classified as 3CP. These results could be due to more severe external surface deviations in 4C and 4Cp types that present with both lumbar and thoracic curves, compared to the 3C types presenting with a single thoracic curve. Although results show that the Schroth exercise program added to the standard care has potential clinical use, a clinically relevant threshold of improvement in asymmetry parameters is not yet known. Further analysis can include comparing perceived changes in appearance by participants and examining changes within specific curve types.

Applying different thresholds for calculating the root mean square (RMS) introduces a limitation that can impact the comparability of results. In order to ensure patch separation and prevent patch overlapping during ST analysis, a standard deviation threshold of 9.33 mm was applied to the majority of the data. However, for ST scans of certain patients where the maximum deviations were below 9.33 mm, it was not feasible to apply the same threshold. In these cases, a reduced normal deviation threshold of 3 mm was utilized instead [16, 17, 19].

Classification results suggest that Schroth exercise treatment has the potential to stabilize or improve scoliosis-related torso asymmetry compared to standard care alone. However, the differences noted were not statistically significant. Results may suggest that a more extended treatment period or a higher exercise dose would have a more substantial effect. A review of early Schroth intervention studies outlines that high amounts of supervised exercises include programs where participants were exercising six days a week for 6–8 hours at a time [31].

Several factors could have resulted in the lack of statistical significance. The improvement threshold of -2.2mm for ΔRMS and ΔMaxDev is unique to this study. Therefore, further work is needed to determine if this threshold is clinical meaningful. Additionally, the progression decision tree was developed to maximize specificity and ensure no cases with progression were missed for use when determining which patient may require a new treatment rather than to detect effects of treatments. Moreover, our sample included patients with less severe curves, which could present a challenge in observing a change over time. Therefore, we recognize that our ST model might not have been sensitive enough to detect meaningful changes in this group of patients with mild to moderate curve magnitudes. Participants classified as presenting progression from ST only have a 30% chance of actual progression [20], and it is possible that participants were incorrectly classified in the progression group.

The effect of offering Schroth exercise during the last six months of the 1-year follow-up in the control group showed no significant difference for both intention to treat and the per protocol analysis. In the control group, attendance of the prescribed supervised sessions was 73%, and the completed home exercises was 68%. The Schroth group attended 76% of the supervised sessions and completed 72% of the prescribed home sessions. The discrepancy in adherence to the treatment between the Schroth group and the control group may have played a role in the observed outcomes. Additionally, participants in the control group with decreased RMS and MaxDev measures (ΔRMS ≤ 0 and ΔMaxDev ≤ 0, n = 12) between the six-months and 1-year follow-ups attended 92% of the supervised sessions and completed 80% of the prescribed home exercises. Participants in the control group with increased RMS and MaxDev measures (ΔRMS > 0 and ΔMaxDev > 0, n = 16) between the six-months and 1-year follow-ups attended 72% of the supervised sessions and completed 71% of the prescribed home exercises. This suggest that adherence to treatment is essential to yield significant results.

Studies that evaluated asymmetry were compared with our RCT. *Kuru et al*. evaluated waist asymmetry of participants randomized into control, home exercises, and supervised exercises groups [13]. Results showed that the supervised group had the most significant decrease between baseline and 24^th^-week measurement. *Schumann et al*.*’s* pilot study measured the average lateral deviation and average surface rotation of participants in different postural positions after 3–4 weeks of intensive rehabilitation [14]. The study showed an improvement of both parameters in the conscious and corrected posture. *Weiss et al*. focused on the effect of the Activities of Daily Living (ADL) rehabilitation approach using ST [15]. Lateral deviation had a greater decrease (non-significant) in the ADL group compared to the control group. Although asymmetry analysis procedures reported in these studies differed from this RCT, the findings support that exercise treatment can have a positive effect on aesthetic measures.

This RCT studied the effect of Schroth exercises added to the standard care, but the effect of Schroth alone cannot be determined. This study could not assess the exclusive impact of Schroth exercises due to their combination with standard care including observation and bracing. To achieve that, patients would need to be randomized into exercise-only and brace-only groups, which is ethically challenging. Our focus was on evaluating the supplementary effect of adding Schroth PSSE to standard care, rather than using it as a standalone treatment. In line with North American standards, involving observation and bracing for patients with curves ≤45°, our sample included an experimental group receiving Schroth in combination with observation or bracing and a control group receiving observation or bracing exclusively. We ensured balanced distribution of observation and bracing in both groups and controlled for brace-related factors in our analyses. The proportions of participants prescribed bracing between the Schroth and control group was relatively balanced, with 65% of participants having received Schroth and wearing a brace, compared to 70% of participants wearing brace only. Groups did not differ significantly for baseline Cobb angle. The average baseline Cobb angle for participants with mild curves (< 25°) in the Schroth group was 18.8° ± 3.8° and 18.1° ± 3.2° in the control group. Likewise, the baseline Cobb angle for participants with moderate curves (≥ 25°) in the Schroth group was 33.3° ± 5.6° and 32.8° ± 5.5° in the control group. Thus, there was a balanced distribution of mild and moderate Cobb angle degrees between the groups.

This RCT did not monitor participants’ brace wear compliance, since brace monitors were not available to us at that time making it difficult to assess differences in bracing compliance between the Schroth and control groups. Ultimately, this RCT aimed to determine the effect of Schroth PSSE added to standard care, not as a standalone treatment. Another limitation was the treatment period of the Schroth exercises, where a minimum short-term treatment of 12 months is recommended to produce reliable results [32]. However, adherence would likely be affected during a longer treatment, and our results show that better outcomes are achieved when participants follow the prescribed protocol. Additionally, this study did not look at whether the change in asymmetry during the six months of the Schroth exercise can be maintained after the supervised treatment is ceased.

Another limitation is the absence of a minimum clinically important difference (MCID) for the ST parameters in this RCT. While, establishing a MCID for the ST parameters was not a focus of this study, future research should establish a MCID to improve the interpretation of clinical relevance of the findings in ST research.

Nevertheless, there are important strengths in this study to highlight. We conducted an RCT, reported both intention to treat and per protocol analyses, and reported an analysis with control group participants serving as their own control after receiving six months of exercises. We also aimed to reduce bias in the results by conducting a blinded analysis of the ST data and classification. We also showed that objective markerless ST asymmetry measurements are sensitive to change over a 6-month Schroth intervention.

## Conclusion

Schroth exercise added to standard care for participants with AIS improved objective surface topography measures of back asymmetry. Both intention to treat and per protocol analyses yielded similar results, but those who completed the trial had a more significant decrease in quantitative asymmetry measurements.

## Supporting information

S1 File CONSORT checklist. (DOC)

S2 File Study protocol for ethics approval. (PDF)

S3 File Linear mixed effects model coefficients, standard error, and significance values for estimating RMS and MaxDev in intention to treat and per protocol analyses. (DOCX)

S4 File Study data. (XLSX)
