# Supplementary material for: Effect of adding Schroth physiotherapeutic scoliosis specific exercises to standard care in adolescents with idiopathic scoliosis on posture assessed using surface topography: A secondary analysis of a Randomized Controlled Trial (RCT)
Source: PLoS One. 2024 Apr 30;19(4):e0302577. doi: 10.1371/journal.pone.0302577 (PMC11060560; doi:10.1371/journal.pone.0302577)
Supplement: S2 File — (PDF) [file pone.0302577.s002.pdf]

1.1 Study Identification

*All questions marked by a red asterisk * are required fields. However, because the mandatory fields have been kept to a minimum, answering only the required fields may not be sufficient for the REB to review your application.*

Please answer all relevant questions that will reasonably help to describe your study or proposed research.

1.0 * **Short Study Title** *(restricted to 250 characters)*:
Multicenter SETS study

2.0 * **Complete Study Title** *(can be exactly the same as short title)*:

Multicenter Schroth Exercise Trial for Scoliosis

3.0 * **Select the appropriate Research Ethics Board (***Detailed descriptions are available at here***):**
HREB Biomedical

4.0 * **Is the proposed research:**
Funded (Grant, subgrant, contract, internal funds, donation or some other source of funding)

5.0 * **Name of local Principal Investigator:**
Eric Parent

6.0 * **Type of research/study:**
Faculty/Academic Staff

7.0 **Investigator's Supervisor***(required for applications from undergraduate students, graduate students, post-doctoral fellows and medical residents to REBs 1 & 2. HREB does not accept applications from student PIs)*:

8.0 **Study Coordinators or Research Assistants:** People listed here can edit this application and will receive all email notifications for the study:

Name	Employer
Kathleen Shearer	MH Surgery
Yibo Li	Student

Name	Employer
Camille Warner	Student
Sanja Schreiber	RM Physical Therapy
Elise Watkins	Student
Courtney Hebert	Student

9.0 **Co-Investigators:** People listed here can edit this application and will receive email notifications *(Co-investigators who do not wish to receive email, should be added to the study email list team below instead of here).*

Name	Employer
Marc Moreau	TEMP
Lindsey Westover	EN Mechanical Engineering
Douglas Hill	MH Surgery

10.0 **Primary Admin Contact:**

11.0 **Study Team:** *(Co-investigators, supervising team, other study team members) - People listed here cannot view or edit this application and do not receive email notifications.*

Last Name	First Name	Organization	Role/Area of Responsibility	Phone	Email
Hedden	Doug	University of Alberta, dept. Surgery	Chair		
Lou	Edmond				
Dinu	Irina				
Tsuyuki	Ross				
Parent	Stefan				

Last Name	First Name	Organization	Role/Area of Responsibility	Phone	Email
Fortin	Carole				
Parsons	David				

1.2 Additional Approval

1.0 * **Departmental Review:** ***Please note only ONE Department Review is required. Please ensure that this section reflects only the PRIMARY Department of the study PI.***

RM Physical Therapy

2.0 **Internal Review** *(If the Principal Investigator is in the Department of Medicine complete the Department of Medicine Request for Internal Approval form and upload it to the "Documentation" section of this application under item 11.0 "Other Documents". Note that all fields in the form are required. The form is available at here)*:

1.3 Study Funding Information

1.0 * **Type of Funding:**
Grant (external)

2.0 * **Indicate which office administers your award.** *(It is the PI's responsibility to provide ethics approval notification to any office other than the ones listed below)*
University of Alberta - Research Services Office (RSO)

To connect your ethics application with your funding: provide all identifying information about the study funding – multiple rows allowed. For Project ID, enter a Funding ID provided by RSO/PeopleSoft Project ID*(for example, RES0005638, G018903401, C19900137, etc).***Enter the corresponding title for each Project ID.**

	Project ID	Title	Grant Status	Sponsor	Project Start Date	Project End Date	Purpose	Other Information
View	RES0050918							
View	RES0004194							
View	RES0017547							SickKids New Investigator Grant NI14-018R

3.0 * **Funding Source**

3.1 Select all sources of funding from the list below:

Glenrose Rehabilitation Hospital	GRH
Scoliosis Research Society	SCOL
Sick Kids Foundation	SICKK

3.2 If your source of funding is not available in the list above, click "Add" below and write the Sponsor/Agency name(s) in the free text box that pops up. *(Nore: You may reflect multiple sources of funding by continuing to click "Add" to add each additional source of funding).*
There are no items to display

4.0 * **Indicate if this research sponsored or monitored by any of the following:**
Not applicable

The researcher is responsible for ensuring that the study complies with the applicable US regulations. The REB must also comply with US Regulations.

1.4 Conflict of Interest

1.0 *** Are any of the investigators or their immediate family receiving any personal remuneration (including investigator payments and recruitment incentives but excluding trainee remuneration or graduate student stipends) from the funding of this study that is not accounted for in the study budget?**

○ Yes ● **No**

2.0 *** Do any of investigators or their immediate family have any proprietary interests in the product under study or the outcome of the research including patents, trademarks, copyrights, and licensing agreements?**

○ Yes ● **No**

3.0 *** Is there any compensation for this study that is affected by the study outcome?**

○ Yes ● **No**

4.0 *** Do any of the investigators or their immediate family have equity interest in the sponsoring company? (This does not include Mutual Funds)**

○ Yes ● **No**

5.0 *** Do any of the investigators or their immediate family receive payments of other sorts, from this sponsor (i.e. grants, compensation in the form of equipment or supplies, retainers for ongoing consultation and honoraria)?**

○ Yes ● **No**

6.0 *** Are any of the investigators or their immediate family, members of the sponsor’s Board of Directors, Scientific Advisory Panel or**

comparable body?

○ Yes ● **No**

7.0 * **Do you have any other relationship, financial or non-financial, that, if not disclosed, could be construed as a conflict of interest?**

○ Yes ● **No**

Please explain if the answer to any of the above questions is Yes:

Important

If you answered YES to any of the questions above, you may be asked for more information.

ID: Pro00043397 **Pro00043397**

Status: Approved 1.5 Research Locations and Other Approvals

1.5 Research Locations and Other Approvals

1.0 * **List the locations of the proposed research, including recruitment activities. Provide name of institution, facility or organization, town, or province as applicable**

EDMONTON SITE:

University of Alberta Hospital - Scoliosis Clinic (Recruitment, and initial eligibility assessment)
University of Alberta- Clinical Sciences Building, room 6-107 Scoliosis Surface Topography Lab
University of Alberta - Rehabilitation Sciences - clinical Sciences building 6-107, Principal Investigator's Lab(Assessments, treatment)
Primary care physicians in Edmonton area (to begin recruitment by asking physicians to refer patients to us.
EPICORE - Clinical trial coordination centre (Scheduling of exams, randomization services and data management)

Montréal
Hôpital Sainte-Justine, Montréal,
Hôpital Marie-Enfant, Montréal
Primary care physicians in the Montréal area (to begin recruitment by asking physicians to refer patients to us.)

2.0 *** Indicate if the study will use or access facilities, programmes, resources, staff, students, specimens, patients or their records, at any of the sites affiliated with the following** *(select all that apply):*
Alberta Health Services Institutions and Facilities

List all health care research sites/locations:
EDMONTON SITE
Recruitment will primarily be done via the Edmonton Scoliosis Clinic at the Glenrose Rehabilitation Hospital and Stollery Hospital (Radiographs and Quality of Life questionnaires will be extracted from the clinical databases). In addition, the primary care physicians will also be involved in recruitment should they agree to.
Surface topography scans will be recorded at the Clinical Sciences Building, room 6-107 Scoliosis Surface Topography Lab, affiliated with the Stollery's Edmonton Scoliosis Clinic.

Montréal.
In Montréal the sites above are not linked with AHS or Covenant Health.

3.0

Multi-Institution Review

*** 3.1 Has this study already received approval from another REB?**
○ Yes ● No

4.0 **If this application is closely linked to research previously approved by one of the University of Alberta REBs or has already received ethics approval from an external ethics review board***(s)***, provide the study number, REB name or other identifying information. Attach any external REB application and approval letter in the Documentation Section – Other Documents.**
The present study is an extension to multiple site of the pilot SETS study approved by HREB as Pro00011552 with the exception that we do not plan to collect blood samples at this point in the multicenter study.

- The surface topography assessment protocol has been used in the study:"Full Torso Surface Topography for Scoliosis" (PRO00006268) and in the study:"360 Degrees Surface Topography Parameters (Norms)" (PRO00003600)
- The use of SRS-22 Questionnaire was approved in all of the above mentioned studies.
- The physical assessment has been approved in the PRO00006957 and

in the PRO00003600 studies.
- The radiograph analyses have been approved in the PRO00006268 study.

2.1 Study Objectives and Design

1.0 * **Provide a lay summary of your proposed research which would be understandable to general public**

Summary

Adolescent Idiopathic Scoliosis (AIS), a 3D spinal deformity, is the most common orthopedic condition leading to elective surgeries in adolescents and affects mostly females. Scoliosis reduces quality-of-life, often results in pain, poor function and self-image.
The Schroth method utilizes asymmetric 3D endurance, strength and breathing exercises aiming to correct posture and scoliotic curves.

A systematic review on scoliosis exercises found that assymmetric exercises slowed the worsening of scoliosis. Many cohort studies showed that Schroth method could be effective in slowing the curve progression, as well as improving the scoliotic curves. Slowing scoliosis progression may help prevent invasive surgery and bracing.

However, history showed that not all patients benefited from Schroth exercises. A strong RCT is needed to confirm results from cohort studies and to identify the predictors of success in patients with AIS treated with Schroth exercises.

A total of 208 female patients with juvenile idiopathic scoliosis (JIS), AIS, or syringomyelia without chiari , curves 10°-45°, between the ages of 10-16, with all Schroth curve types (3c, 3cp, 4c and 4cp) and Risser sign less or equal to 3 will be recruited from specialized Scoliosis clinics in Edmonton, Calgary and Montréal. Patients will be

randomized to observation/bracing(as the current treatment standard dictates) or Schroth exercises (alone or with brace). History, questionnaires, physical, and surface topography assessments will be done at baseline, 3 and 6 months. Radiographs and SRS-22 questionnaires will be extracted from the Clinic's database, at baseline, 6 and 12 months and every 6 months until discharge from the scoliosis clinic. The control group will receive no additional treatment. The Schroth exercise program will consist of 5 individual sessions, followed by weekly group sessions and a home exercise program for 6 months. A certified Schroth therapist will prescribe exercises using an algorithm. After 6 months control subjects will crossover to the control group. Recruitment, eligibility, compliance, and drop out rate, along with effect sizes for all outcomes will be determined. Intent-to-treat linear mixed-models will be used to compare the effects between groups over time while controlling for covariables.

2.0 * **Provide a full description of your research proposal outlining the following:**

- **Purpose**
- **Hypothesis**
- **Justification**
- **Objectives**
- **Research Method/Procedures**
- **Plan for Data Analysis**

Schroth Exercise Trial for Scoliosis

Background

Adolescent Idiopathic Scoliosis (AIS), a 3D spinal deformity, is the most common orthopedic condition in adolescents requiring elective surgery and affects mostly females. In mild scoliosis from 10° to 25° the ratio is 4:1, in moderate AIS from 25° to 50° the ratio is 7:1, while in severe AIS $>50^{o}$ the ratio is 10:1. Scoliosis reduces quality-of-life, often results in pain, poor function and self-image.

Usually, curves between $25\text{-}45^{o}$ are prescribed a brace and curves over 45° are treated surgically. Smaller curves are not treated. The

Schroth method utilizes asymmetric 3D endurance, strength and breathing exercises aiming to correct posture and scoliotic curves. Schroth therapists provide support by skillful resistances, auxiliary handholds, and specific verbal instructions and feedback from mirrors. Schroth exercises aim to restore proper physiological three-dimensional alignment.

Schroth treatment has proven effective in improving strength, posture, slowing progression and improving Cobb angle in many cohort studies. However, these results have not yet been tested in RCTs.

Objectives

To conduct a RCT on the effect of Schroth exercises for AIS compared to the current treatment standard (observation or bracing prescribed as per SRS guidelines). Specific objectives are to compare the effects of 6-month of Schroth exercises added to standard care to standard care alone on curve severity measured using the Cobb angle, and on secondary outcomes including quality-of-life, spinal appearance, objective posture and spinal muscle endurance measurements. We will also compare the effect of 6 months of Schroth therapy offered to control participants after completing 6 months in the standard care group and determine if effects can be maintained until maturity once supervision is terminated.

Methods

<u>Subjects</u>

Female patients with AIS, JIS, or syringomyelia without chiari (n=208) will be recruited in Edmonton, Calgary and Montréal. Inclusion criteria are: (1) curves 10°-45°, (2) age 10-16 (3) All Schroth curve types (3c, 3cp, 4c and 4cp), (4) treated with or without brace (5) living within 1 hour of the study site and (6) with Risser sign less than or equal to 3. Those planning or post-surgery will be excluded.

<u>Procedures</u>

At routine scoliosis clinic visits a nurse will determine eligibility and randomize participants to the observation (control) or the Schroth

exercise group. Outcomes will be measured at baseline, 3 and 6 months. Primary outcomes include baseline and 6-month radiographic curve measurements and SRS-22 questionnaire. Secondary outcomes will include postural measures (Surface Topography), Global Rating of Change (GRC), Self-efficacy questionnaire, pain, and back muscle endurance. Predictors of success will be collected at baseline.

Controls will receive the current standard of treatment for 6 months (Observation or brace prescribed as per SRS guidelines). Patients treated with Schroth exercises will participate weekly in supervised Schroth exercises and complete a daily home program for 6 months. The baseline and 6-month exam will be completed within 2 weeks of a scoliosis clinic visit with a follow-up at 3 months. After 6 months, controls will crossover to the treatment group. After 12 months and for every scoliosis visit afterwards until discharge, we will also extract the radiograph and SRS-22 questionnaire from the Edmonton Scoliosis records

<u>Schroth exercises:</u>

Patients for which the SRS guidelines would recommend observation as standard of care will do exercises alone. Patients for which the SRS guidelins would recommend bracing will do exercises in addition to being prescribed a brace.

Private sessions- During the first 2 weeks, patients will attend 5 1-hour-long individual sessions to learn Schroth principles, exercises and correct breathing, and to confirm independent adequate performance with a checklist.

Home exercises- A 30-min. daily home exercise program of 3 to 4 exercises will be adjusted during the first 6 months using an algorithm. Compliance will be monitored using a logbook for 6 months. Completion exercises during the last 12 months will also be monitored using a logbook.

Group Sessions- Classes increase compliance. Patients will attend weekly 1-hour-long group therapist-led exercise classes. Adequate

exercise performance will be assessed using the checklist. Pain will be monitored.

Algorithm – An algorithm guides exercise prescription, intensity and progression from static to dynamic execution. Prescription begins with easier exercise. If performed adequately, a more challenging exercise will be tried. If performance is inadequate, an easier exercise will be attempted. Exercises performed adequately will be prescribed.

At follow-up, if performance is adequate, dosage will increase to target intensity. Target intensity increases repetitions aimed to enhance endurance. Final tension intensity is nearly maximum isometric contraction held in corrected posture.

During the COVID-19 pandemic, the few participants still receiving exercises will be offerred the choice to receive therapy in person or via videoconference. Our therapist Graham has access to a secure videoconferencing system (JaneApp) to deliver this therapy remotely. Alternatively Dr Parent has access to a secure ZOOM space. No recording is made during the videoconferencing session.
.

<u>Controls:</u> Controls will receive the standard of care appropriate for their curves (observation or bracing prescribed as per SRS guidelines), and will complete baseline, 3 and 6 months exams. After 6 months they will cross-over to the treatment group.

<u>Questionnaires:</u>

Demographic/History- Socio-demographics, symptoms history and modifying factors will be self-reported.

Pain rating and diagram- A numeric scale from 0 (no pain) to 10 (worst imaginable pain) will assess current, best, worst intensity in the last 24 hours. A diagram will assess location of symptoms.

Scoliosis-Research-Society questionnaire (SRS-22r)- The SRS-22r assesses quality-of-life within 5 domains: function, pain, self-image, mental health (5 questions each), and satisfaction (2 questions).

3D Physical activity recall (3DPAR)- quantifies habitual physical activity chosen among 55 activities listed, and their intensities for blocks of 30-min over 3 days. The 3DPAR score is determined using the metabolic equivalent (MET) levels.

Spinal Appearance Questionnaire- includes 21 items pertaining to the severity of the deformity within 9 domains (Trunk shift, Waist, Kyphosis, Prominence, Chest, Shoulders, General and Curve) .

Self-efficacy – measures self-efficacy for overcoming barriers to physical activity using 8 items rated from 1 (Disagree a lot) to 5 (Agree a lot).

Global Rating of Change (GRC)- A 15-point GRC ranging from –7 (very great deal worse) to +7 (very great deal better) will be used.

Physical Exam*(Appendix E)*: An physiotherapist blind to groupings will assess curve type and hip range-of-motion in flexion, extension, internal rotation, the straight-leg-raise and FABERE tests. Sorensen's test will assess back extensor muscle endurance. Lumbar (inclinometers at L5/S1 and T12/L1) and thoracic (C7/T1 and T12/L1) spinal range-of-motion will be recorded: lumbar flexion, extension and side flexions. Scoliometer will record asymmetry during the Adam bending test.

Radiographs- Standing postero-anterior radiographs in a positioning frame at baseline, 6 and 12 months and from every scoliosis clinic visits until discharge will be used to measure the Cobb angle, rotations, apex translation. An experienced evaluator blind to group assignment will extract measures.

Surface topography (ST) – An experienced evaluator will operate four scanners and use a positioning frame to scan torso shape. The following ST parameters will be extracted by digitizing 15 points on de-identified scans (blinding): cosmetic score, shoulder angle, scapula angle, waist asymmetry, trunk rotation, kyphotic and lumbar indices.

Analyses:

Intention to treat (by carrying forward last known value) linear mixedmodel analyses will be used to assess differences in group changes from baseline, to 3 and 6-month while adjusting for covariates. Separate analyses will be conducted for each outcome. To screen for variables associated with each of the outcomes variables, we will use univariate linear mixed models and include all relevant covariates (age, time since menarche, apex levels, physical activity, self-efficacy, and bracing compliance may have associations with risk of progression). Multiple linear mixed models will be tested including covariables found significant at P=0.20 in the univariate analysis, and after screening for multicolinearity. Results will be presented as effect sizes with confidence intervals and one-sided P-values. Statistical analyses will be performed using SAS version 9.1.

For subjects initially in the control group later completing 6 months of exercises, intention to treat linear mixed-model analyses will be used to assess differences between the two periods (standard care vs standard care + Schroth) in changes from baseline, to 3 and 6-month, while adjusting for covariates. Screening for covariates will be done as described above. In this model, treatment period is a dependent factor.

To determine if exercises effects are maintained at 12 months and discharge exams, linear mixed- model analyses will also be used to quantify the effect sizes corresponding to changes from 6 to 12 months and to discharge by combining data from all patients after participating in the exercise program.

3.0 Describe procedures, treatment, or activities that are above or in addition to standard practices in this study area *(eg. extra medical or health-related procedures, curriculum enhancements, extra follow-up, etc)***:**

SRS-22 and Spinal appearance questionnaires, surface topography and radiograph scanning (which will be extracted from the hospital database) are standard practices at the specialized Scoliosis Clinic. The data routinely collected at scoliosis clinic visits will be used in the study. Clinics are routinely scheduled every 6 months for patients meeting the eligibility criteria in our study.

Our 3 months assessment is additional to the routine visits. Some tests at baseline, 3 and 6 months are additional to routine visits and all exercise treatments are additional to routine visits.

Participating in the study requires completing tests and questionnaires in addition to usual procedures. Physical examination at baseline, 3 and 6 months including range of motion of back and lower extremity are not current practice. The following questionnaires completed at baseline, 3 and 6 months are extra procedures, too: Demographic/History, Pain rating and diagram, 3D Physical activity recall, Self-efficacy and Global Rating of

Change.

All exercise visits are additional to routine scoliosis visits.

4.0 **If the proposed research is above minimal risk and is not funded via a competitive peer review grant or industry-sponsored clinical trial, the REB will require evidence of scientific review. Provide information about the review process and its results if appropriate.**
Glenrose Hospital Foundation,Clinical Research Grant Review Committee (completed)
Scoliosis Research Society, Small Exploratory Grant Review Committee (completed 2009 and 2010)
Women's and Children Health Research Institute, CIHR Pre-review Process (completed)
SickKids Foundation New Investigator Review committee (awarded in 2013, completed also in 2010)

5.0 **For clinical trials, describe any sub-studies associated with this Protocol.**

2.2 Research Methods and Procedures

Some research methods prompt specific ethical issues. The methods listed below have additional questions associated with them in this application. If your research does not involve any of the methods listed below, ensure that your proposed research is adequately described in Section 2.1: Study Objectives and Design or attach documents in the Documentation Section if necessary.

1.0 * **This study will involve the following***(select all that apply)*
Surveys and Questionnaires (including internet surveys)
Radiation: Any test or procedure that may involve exposure to radiation (including screening chest x-ray)

NOTE 1: Select this if you are directly collecting health information as part of your protocol OR will be conducting a chart/record review/reviewing health data secondarily. This includes anonymized or identifiable health information.

NOTE 2: Select this option if this research ONLY involves analysis of blood/tissue/specimens originally collected for another purpose but now being used to answer your research question. If you are enrolling people

into the study to prospectively collect specimens to analyze you SHOULD NOT select this box.

NOTE 3: This section is intended to reflect the secondary use of non-health data. Do NOT select this if you are using data that originally came from health sources, i.e., anonymized administrative data.

2.9 Surveys and Questionnaires (including Online)

1.0 **How will the survey/questionnaire data be collected (i.e. collected in person, or if collected online, what survey program/software will be used etc.)?**
Participants will be asked to complete questionaires at home and bring them to the appointment with them.

2.0 **Where will the data be stored once it's collected (i.e. will it be stored on the survey software provider servers, will it be downloaded to the PI's computer, other)?**
Paper in locked file cabinet in room 6-107 and entered in REDCAP.

3.0 **Who will have access to the data?**
PI and study team members.

4.0 **If you are using a third party research tool, website survey software, transaction log tools, screen capturing software, or masked survey sites, how will you ensure the security of data gathered at that site?**

2.20 Radiation Safety

1.0 **Will your research involve any of the following?** *(Check all that apply)*
X-rays of the skull, facial bones, neck, spine, thorax, abdomen, pelvis or hip

2.0 **Research involving exposure of participants 0-17 years of age to any amount ionizing radiation, regardless of how little, must be approved by the AHS Regional Radiation Safety Committee (RSC). Will your research involve exposure to participants aged 0-17 years to any amount of ionizing radiation?**

● **Yes** ○ No

Please describe

3.0 **If this application is for the amendment of a pre-existing clinical study, have procedures which involve exposing subjects to ionizing radiation been added to the research that was not identified in the original study protocol?**

Note: If you answered YES to any of the above, the system will forward your project information to the AHS Regional Radiation Safety Committee for review. You will be notified of any issues pertaining to RSC approval which may include adding a radiation risk statement to the patient information sheet/consent form or the rewording of an existing risk statement. Protocol amendment is rarely necessary.

For further information, contact the RSC by email at radnsfty@ualberta.ca.

Gail Schaffler, MRT (R)
Research Technologist

Dr. Derek Emery
Professor and Chair
Department of Radiology and Diagnostic Imaging
The University of Alberta

ID: Pro00043397 **Pro00043397** 3.1 Risk Assessment
Status: Approved

3.1 Risk Assessment

1.0 * **Provide your assessment of the risks that may be associated with this research:**
Minimal Risk - research in which the probability and magnitude of possible harms implied by participation is no greater than those encountered by participants in those aspects of their everyday life that relate to the research (TCPS2)

2.0 * **Select all that might apply:**

Description of Possible Physical Risks and Discomforts	
Possibly	Participants might feel physical fatigue, e.g. sleep deprivation
No	Participants might feel physical stress, e.g. cardiovascular stress tests
Possibly	Participants might sustain injury, infection, and intervention side-effects or complications
Possibly	The physical risks will be greater than those encountered by the participants in everyday life

Possible Psychological, Emotional, Social and Other Risks and Discomforts	
Possibly	Participants might feel psychologically or emotionally stressed, demeaned, embarrassed, worried, anxious, scared or distressed, e.g. description of painful or traumatic events
Possibly	Participants might feel psychological or mental fatigue, e.g intense concentration required
Possibly	Participants might experience cultural or social risk, e.g. loss of privacy or status or damage to reputation
No	Participants might be exposed to economic or legal risk, for instance non-anonymized workplace surveys
Possibly	The risks will be greater than those encountered by the participants in everyday life

3.0 * **Provide details of all the risks and discomforts associated with the research for which you indicated YES or POSSIBLY above.**
- For physical assessment:
We do not expect adverse effects to result from the study physical assessment. All the procedures are routinely used in the assessment of patients with scoliosis and are considered non-invasive. In the unlikely event where we would detect a spinal deformity warranting further investigation in the otherwize healthy teenagers, we will provide

information on the nature of our findings and suggest referral to the patient’s family physician. We do not expect that subjects will suffer any side-effects from the physical assessment in this study. The assessment involves simple movements and tests. Participants should not feel pain in this study. Rarely, participants may feel sore during the first few days following a simple physical assessment. For those experiencing soreness it should disappear in a few days. The tests are not invasive and do not use radiation. There could be other side-effects which it is not possible to anticipate.

- For the treatment:
Schroth exercises are designed to improve the ability to maintain correct posture through endurance and strength training. Schroth exercises include de-rotation, de-flexion, and stretching exercises to establish vertebral alignment, torsional respiration exercises, and strengthening exercises for the abdominal, back, leg and foot muscles, and as such are not expected to have any adverse effects on patients. The exercises involve simple movements and positioning. Participants should not feel pain in this study. Rarely, participants may feel sore during the first few days following trying a new exercise. For those experiencing soreness it should disappear in a few days.
- For the surface topography:
The full torso scanning procedure involves removing clothes of the torso, hiding the breast using stick-on bra and having some reference points marking on the skin. Some participants may feel uncomfortable with their torso exposed.
-In both study groups, some patient's scoliosis may progress to the extent where bracing or surgery prescription criteria are met.

Due to the COVID-19 pandemic, there is a possibility of infection transmission with additional visits to the lab and human interactions the context of the research.

4.0 * **Describe how you will manage and minimize risks and discomforts, as well as mitigate harm:**
- We do not expect adverse effects to result from the study. All the procedures are routinely used in the assessment of patients with scoliosis and are considered non-invasive. In the unlikely event where we would detect a spinal deformity or a pathology warranting further investigation, we will provide information on the nature of our findings and suggest referral to the patient’s family physicians. We will be available to discuss our findings with the physician at the patient’s request. Testing procedure can be interrupted at the patient's request at anytime.

- The surface topography scanning procedures are all done by a female. The subjects will be oriented to the scanning area. Participants will be instructed in the use of the stick-on bras. The evaluator will then leave the patient in the closed off scanning area to disrobe privately and apply the bras. The patient will be given a hospital gown for privacy when the

evaluator returns to apply the reference marks on the skin and to adjust the positioning frame. The evaluator will then leave again and the patient will disrobe. The surface scan will be obtained immediately. During the scan the subject will be offered to be accompanied by a family member or a friend if they desire. The scanner operator will be a female. The analysis of the scan will be done by a female and the head will be cropped off of the scan to protect patient privacy. Patients may refuse to participate at any point.

- Schroth therapist will individually instruct all the patients in the execution of the exercises in the first 5 sessions, and the group sessions will be led by a Schroth therapist. All exercises can be modified to minimize possible discomfort that can be caused by inadequate level of intensity.

- In the case of patient's scoliosis progression to the extent where bracing or surgery prescription criteria are met, they will be referred to their treating surgeon and prescribed treatment deemed appropriate. SRS prescription criteria will be used.

For exams, participants will be invited to complete questionnaires at home to minimize visit duration. All exams will occur in CSB to avoid visiting too many sites which would increases their risk of exposure to COVID 19. The EHS plan for Dr Parent's lab requires a clear AHS Covid-19 screen for both patients and therapist.
For patients the requirements are as follows:strict control of building access, not allowing accompanying person access in the lab unless assistance is needed, patients are required to wear disposable mask provided by the lab, and patients are required to wash their hands before and after the exam.For the therapist, requirements are as follow: required to wear disposable mask, visor, gown and gloves, have a clear Covid 19 screen and wash hands before and after each patient.
Our evaluation equipment and clinic room will be cleaned and disinfected as per our EHS return to work plan before and after every patient. We will space exams at least one 1 hour apart to allow time for this cleaning and minimize interactions between different people accessing the lab. The number of persons in the lab is tightly controlled with a maximum of 3 including patient for the exam (with routine generally being only the examiner and the patient).

The few participants receiving therapy will be given a choice to come to CSB for therapy in person (individual (not groups)) or online via secure videoconference.

5.0 **Is there a possibility that your research procedures will lead to unexpected findings, adverse reactions, or similar results that may require follow-up** *(i.e. individuals disclose that they are upset or distressed during an interview/questionnaire, unanticipated findings on*

*MRI, etc.)***?**

◯ Yes ● **No**

6.0 **If you are using any tests in this study diagnostically, indicate the member(s) of the study team who will administer the measures/instruments:**

Test Name	Test Administrator	Organization	Administrator's Qualification

There are no items to display

7.0 **If any research related procedures/tests could be interpreted diagnostically, will these be reported back to the participants and if so, how and by whom?**

3.2 Benefits Analysis

1.0 * **Describe any potential benefits of the proposed research to the participants. If there are no benefits, state this explicitly:**
There are possible benefits for the participants in this study. There is evidence that suggests Schroth exercises can improve spinal curvature, slow the progression of scoliosis, correct posture, and increase strength in some patients with AIS. The benefits are not guaranteed and patients that have received Schroth exercise treatment in the past have not always had these results. If findings from the evaluations suggest that you need additional medical attention, you will be contacted and referred appropriately.

2.0 * **Describe the scientific and/or scholarly benefits of the proposed research:**
- Results of our study will help determine whether this exercise treatment is appropriate to implement at the Edmonton Scoliosis clinic as an alternative to standard practice (observation/ or bracing) for the smaller curves (10-45 degrees according to Cobb).
- Results of our study may also help other clinics in the world to decide whether they want to offer Schroth treatment.

3.0 **If this research involves risk to participants explain how the benefits outweigh the risks.**
Risks are minimal and controlled. The results could potentially have great

benefit for clinical practice by providing evidence for the conservative treatment for adolescent idiopathic scoliosis and assist physicians in prescribing exercises to the right patient.

With our careful mitigation procedures for COVID-19 risk, the benefits are potentially important.

4.1 Participant Information

1.0 * **Will you be recruiting human participants** *(i.e. enrolling people into the study, sending people online surveys to complete)***?**

● **Yes** ○ No

1.1 Will participants be recruited or their data be collected from Alberta Health Services or Covenant Health or data custodian as defined in the Alberta Health Information Act?

● **Yes** ○ No

1.2 Would you like to include information about this study on the Be The Cure searchable database?

○ Yes ○ No

4.2 Additional Participant Information

1.0 **Describe the participants that will be included in this study. Outline ALL participants** *(i.e. if you are enrolling healthy controls as well):*
Patients with Adolescent Idiopathic Scoliosis (AIS), with juvenile idiopathic scoliosis (JIS), or syringomyelia without chiari from the Specialized Scoliosis Clinic in Edmonton, Calgary, and Montréal (n=208 total). They are between 10 and 16 years old.

2.0 * **Describe and justify the inclusion criteria for participants** *(e.g. age range, health status, gender, etc.):*

Patients with Adolescent Idiopathic Scoliosis (AIS), with juvenile idiopathic scoliosis (JIS), or syringomyelia without chiari from the Specialized Scoliosis Clinic in Edmonton, Calgary, Montréal(n=208) will be recruited from our scoliosis clinics. AIS is defined as idiopathic scoliosis diagnosed between the ages of 10-16. JIS is defined as idiopathic scoliosis diagnosed between the ages of 5-10. Idioapthic scoliosis may have appeared between the ages of 5-10, but not been diagnosed until after the age of 10. Therefore, including patients with JIS and AIS in the study is not including two distinct forms of scoliosis, rather a continuum of idioapthic scoliosis. Patients with syringomyelia without chiari are common and does not exclude a patient's diagnosis as AIS or JIS.

Only females are targeted. Females are more frequently severely affected to levels justifying a treatment prescription. Other inclusion criteria are:

(1) magnitude of spinal curve 10° to 45° (Cobb angle),

-Larger curves meet the clear indication recommended by the Scoliosis Research Society to be prescribed surgery. Small curves in skeletally immature adolescents have higher risk of progression. Curves over 30° are more likley to progress after skeletal maturity. European consensus for conservative treatment recommends exercises alone below 20° and exercises in combination with a brace for patients with curves greater than 20°.

(2)may be treated with or without brace

-We have broadened inclusion criteria to be inclusive of all conservative treatment offered at the Specialized scoliosis clinic in Edmonton, Calgary and Montréal (observation and bracing). European consensus for conservative treatment recommends exercises alone below 20° and exercises in combination with a brace for patients with curves greater than 20°. Including patients with and without brace treatment allows for comparison of exercise alone and in combination with brace treatment. Including brace treatment as a potential predictor of treatment success is representative of decisions clinicians are making in the treatment of patients with scoliosis. We will examine whether bracing in combination with exercises is predictive of success beyond the other treatment combinations.

(3)Risser 0-3 (indicating Incomplete skeletal maturity)

-Patients with curves over 30° have a risk of progression beyond skeletal maturity. Schroth exercises are recommended for all curve sizes.

(4) age 10-16

-Patients are treated between the ages of 10-18 at the Edmonton Scoliosis Clinic. Inclusion of patients over the age of 10 allows for comprehension of Schroth exercise program. Limiting upper limit of age range to 16 increases the likelihood that the patients are skelettally immature and therefore still trully at risk of progression. Patients having reached the skelettal maturity are simply given a recommendation to exercises but the prescription would be deemed optional in most cases.

The SickKids reviewers requested we enroll skeletally immature patients only.

(5) ability to travel to study sites at least weekly.

- To ensure that we can assess the efficacy of the proposed exercise protocol we require the participant to attend weekly exercise sessions at the study sites. This will ensure feasibility of the trial with opportunity for maximal compliance.

3.0 Describe and justify the exclusion criteria for participants:

(1)Patients with curves >45° may be surgical candidates, and will be excluded.

-Our clinic and the Scoliosis research foundation clearly recommend that patients with such large curves should be considered for surgery. In practice, these patients may be prescribed surgery and not deemed candidate for an exercise program.

(2) Patients who have had surgery will be excluded.

-Previous surgical intervention could have a confounding effect in the efficacy analysis.

-A separate study could examine the effect of exercises combined with surgical correction but the current study will not address this question.

(3) Patients under the age of 10 will not be included, as the Schroth exercise program is not designed for juveniles. The level of comprehension may be too difficult for ages 5-10 (age of diagnosis with JIS). Patients over the age of 16 are excluded as treatment and observation at the Edmonton Scoliosis clinic is complete at age 18 and patients over 16 years old a likely to have reached skelettal maturity and in that group exercises prescription would be optional and target a different goal than simply stopping curve progression.

4) Patients with Risser signs in excess of 3 are deemed skelettally too mature to use exercises to slow progression. The exercises may be used in such a group but the goal would be different and does not correspond to the rationale that may convince surgeons managing scoliosis in North America to prescribe intensive exercise programs in patients with scoliosis.

4.0 Participants

4.1 How many participants do you hope to recruit *(including controls, if applicable?)*
100

4.2 Of these, how many are controls, if applicable?
26

4.3 If this is a multi-site study, how many participants do you

anticipate will be enrolled in the entire study?
208

5.0 Justification for sample size:
Primary and secondary aim 1: A sample of 129 subjects per group will be sufficient to detect a 0.31 effect size when comparing the change in outcomes between the exercise and control groups with 80% power using a unilateral test and an alpha of 0.05 (104 Schroth and 104 controls in this study + 25 Schroth and 25 controls in the pilot allows a total of 129 per group). We anticipate >80% completion rate based on published data and because the pilot dropout rate was <10%. Our per-protocol sample may drop slightly however, we are planning intent to treat analyses where we will carry-forward the last known observation from our participants.

A unilateral hypothesis is justified, for this superiority trial, by positive published data and our pilot data. The pilot effect size for the Cobb angle was 0.32 and many secondary outcomes had effect sizes exceeding what we are powered to detect as significant. A trial conclusive in favour of exercises would have a significant impact in America. A trial failing to demonstrate positive results would have less impact as Schroth exercises are not currently prescribed. We believe that a 0.32 effect size is clinically important, feasible, and would justify implementing a Schroth program. The trial duration is relatively short which justifies aiming to detect a small effect size (0.2-0.5) .

It would be inadequate to power the trial to detect too small an effect size as patients and families will likely demand evidence of clinically important effects before committing efforts and time required of Schroth exercise programs. For the secondary aim comparing the results of subjects in the control group who first complete 6 months as controls to their results after completing 6 months of Schroth exercises, an effect size of 0.27 (n=106) could be detected as significant with a power of 0.80, an alpha of 0.05 with a unilateral test.

4.3 Recruitment of Participants (Health)

1.0 Recruitment

* **1.1 How you will identify potential participants? Please be specific.** *(i.e. Will you be screening clinical lists, accessing electronic health records (e-clinician), asking staff from a particular area to let you know when a patient meets criteria, will you be sitting in the emergency department waiting room, etc?)*
At the regular specialized scoliosis clinic at each site, the clinic's nurse will determine the eligibility of each patient and refer a patient to our research coordinator for more information on the study. If a patient decides to participate, an informed consent will be obtained. The contact information of eligible and consented patients will be forwarded to the researchers, who will coordinate with patients from that point on.
Primary care physicians identifying potential candidates will suggest these patients contact the PI to be informed of the study. Interested such participants will be referred to the specialized scoliosis clinic to confirm eligibility and begin participation.

1.2 If you are using patient/clinical records to identify potential participants for research purposes, will someone from the data custodian/clinical care team seek prior consent of the participant to allow the researcher to look at their records?

○ Yes ○ No

1.3 Once you have identified a list of potentially eligible participants, indicate how the potential participants' names will be passed on to the researchers AND how will the potential participants be approached about the research.

1.4 Outline any other means by which participants could be identified*(e.g. response to advertising such as flyers, posters, ads in newspapers, websites, email, list serves, physicial or community organization referrals):*

2.0 Pre-Existing Relationships

2.1 Will potential participants be recruited through pre-existing relationships with researchers *(e.g. Will an instructor recruit students from his classes, or a physician recruit patients from her practice? Other examples may be employees, acquaintances, own children or family members, etc)***?**

● **Yes** ○ No

2.2 If YES, identify the relationship between the researchers and participants that could compromise the freedom to decline *(e.g. clinician/patient, professor/student):*
Some of the study team members are involved in the management of patients attending the site's specialized Scoliosis Clinics. Patients with scoliosis from the sites' specialized Scoliosis Clinic will be informed of the

study and invited to participate by the clinic coordinator when arranging clinic visits. The clinic coordinator will send the study information by email or by phone when booking the clinic visit. The booking is not dependent of patient's agreement to participate. Once informed, patients will contact the researcher to learn more about the project and arrange participation in the study. The coordinator will explicitly mention that patient may refuse to participate. The surgeon may answer questions about the project during the patient's visit but will not be directly involved in the enrollment process. The clinic's nurse practitioner will assist in determining patient's eligibility but will not decide if a patient is ultimately eligible or not (this will be confirmed during the baseline evaluation).

2.3 How will you ensure that there is no undue pressure on the potential participants to agree to the study?

3.0 **Will your study involve any of the following** *(select all that apply)* **?**
Reimbursement for any expenses incurred by the participants, e.g. parking costs, child care, lost wages, etc

4.5 Informed Consent Determination

1.0 **Describe who will provide informed consent for this study** *(i.e. the participant, parent of child participant, substitute decision maker, no one will give consent – requesting a waiver)*

1.1 Waiver of Consent Requested
If you are asking for a waiver of participant consent, please justify the waiver or alteration and explain how the study meets all of the criteria for the waiver. Refer to Article 3.7 of TCPS2 **and provide justification for requesting a Waiver of Consent for ALL criteria (a-e)**

1.2 Waiver of Consent in Individual Medical Emergency
If you are asking for a waiver or alteration of participant consent in individual medical emergencies, please justify the waiver or alteration and explain how the study meets ALL of the criteria outlined in Article 3.8 of TCPS2 (a-f)**.**

2.0 **How will consent be obtained/documented? Select all that apply**

Signed consent form
Verbal consent

If you are not using a signed consent form, explain how the study information will be provided to the participant and how consent will be obtained/documented. Provide details for EACH of the options selected above:
Since we have completed enrollment only participants in need of follow-up will need to have a consent amendment to continue their participation.

Participants will be called prior to appointments and will be advised that the risk involved with this study has changed due to increased possibility for exposure to COVID-19. Participants will be read the telephone script (added in documentation) and will be asked if they would like to continue to be enrolled in this study. Should they agree to continue to participate, we will sign that we have their verbal consent. Participants will be provided with a copy of this verbal consent at their next in person visit.

3.0 Will every participant have the capacity to give fully informed consent on his/her own behalf?

○ Yes ○ No

3.1 Explain why participants lack capacity to give informed consent *(e.g. age, mental or physical condition, etc.).*
Participant will be considered as unable to give informed consent because all enrolled are expected to be younger than 17 years. Since enrolling in the study will require parental support (driving to treatments for up to 6 months, assent from patients and parental consent are the ideal informed consent strategy for this study.

3.2 Will participants who lack capacity to give full informed consent be asked to give assent?

● **Yes** ○ No

Provide details. IF applicable, attach a copy of assent form(s) in the Documentation section.
Assent forms will be provided by email (of fax as needed) prior to the visit and signed during the visit after discussing the study with the coordinator. Opportunities to ask question by phone or before beginning study procedures will be available.

3.3 In cases where participants (re)gain capacity to give informed consent during the study, how will they be asked to provide consent on their own behalf?

We do not anticipate that many patients will be followed-up until 18 years old when they would be able to consent on their own. If they do, we will have them complete a consent form once they reach the age of consent. Nevertheless, all subjects will complete an assent form to ensure that they are informed of the nature of what is expected of them in the study.

4.0 **What assistance will be provided to participants or those consenting on their behalf, who may require additional assistance?** *(e.g. non-English speakers, visually impaired, etc.)*
Researchers will be available to answer question on site when the consent/assent form is signed. Forms will be emailed ahead of participating to ensure the participants have plenty of time to digest the information. The researchers could be contacted via phone or email to answer questions ahead of participation if needed. All subject are under 18yo and will be invited to provide assent and parental consent will be obtained.

5.0 * **If at any time a PARTICIPANT wishes to withdraw from the study or from certain parts of the study, describe when and how this can be done.**
Participants may do so at any time.

6.0 **Describe the circumstances and limitations of DATA withdrawal from the study, including the last point at which participant DATA can be withdrawn** *(i.e. 2 weeks after transcription of interview notes)*
Data withdrawal is possible by patient request at anytime before the analyses are completed.

7.0 **Will this study involve any group(s) where non-participants are present? For example, classroom research might involve groups which include participants and non-participants.**
○ Yes ● **No**

ID: Pro00043397 **Pro00043397**
Status: Approved 4.6 Expense Reimbursements and Incentives

4.6 Expense Reimbursements and Incentives

1.0 **Expense Reimbursements:**

1.1 Describe in detail the expenses for which participants will be reimbursed, the value of the reimbursements per item as well as the total maximum reimbursement and the reimbursement process *(e.g. participants will receive a cash reimbursement for parking at the rate of $12.00 per visit for up to three visits for a total value of $36.00)*
Patients will be asked to commit time for the assessments, the treatments (individual and weekly group classes), and the home exercise routine. No funds are available to compensate patients for their time. Evaluations and treatments are provided at no charge to patients or their insurance. Transportation to the evaluations and treatments are the responsibility of the patients/ parents and will not be reimbursed.

Participants will be provided with parking coupons for each visit to our sites.

1.2 If you will be collecting personal information to reimburse or pay participants, describe the information to be collected and how privacy will be maintained.
Not applicable.

2.0 Incentives:

2.1 Will participants receive any incentives for participating in this research *(i.e. gift card, cash payment, prize draw)***? If yes, provide details of the value, including the likelihood (odds) of winning for prize draws and lotteries.***The Use of Incentives In Research*

Patients will be asked to commit time for the assessments, the treatments (individual and weekly group classes), and the home exercise routine. No funds are available to compensate patients for their time. Evaluations and treatments are provided at no charge to patients or their insurance. Transportation to the evaluations and treatments are the responsibility of the patients/ parents and will not be reimbursed. Participants will be provided with parking coupons for each visit to out sites.

2.2 What is the maximum value of the incentives offered to an individual throughout the research?

2.3 If incentives are offered to participants, they should not be so large or attractive as to constitute undue influence. Justify the value of the incentives you are offering relative to your study population.
Parking passes are a compensation for a cost the patients would have to incur beyond what is expected of them as part of routine care.

It only covers parking, not time not gaz... therefore it is a minor compensation all things considered.

5.1 Data Collection

1.0 * **Will the researcher or study team be able to identify any of the participants at <u>any stage</u> of the study?**

● **Yes** ○ No

2.0 **Primary/raw data collected will be** *(check all that apply)***:**

Indirectly identifying information - the information can reasonably be expected to identify an individual through a combination of indirect identifers (eg date of birth, place of residence, photo or unique personal characteristics, etc)

3.0 **If this study involves secondary use of data, list all original sources:**

Data from the Specialized Scoliosis database at each site will be extracted as described in the informed consent procedures with the permission of the patient: radiograph measures at baseline, 3, 6, 12 months follow-up, SRS-22 questionnaires at the same times.

4.0 **In research where total anonymity and confidentiality is sought but cannot be guaranteed** *(eg. where participants talk in a group)* **how will confidentiality be achieved?**

Total anonymity is not sought per se. Participants will exercise in groups and get to meet other participants. Participants will be informed of the need to maintain confidentiality of personal information learned in the study. They will also be asked to refrain from informing the evaluator of their study group or of that of other participants.

5.2 Data Identifiers

1.0 * **Personal Identifiers:** will you be collecting - at any time during the study, including recruitment - any of the following *(check all that apply)***:**

Surname and First Name

Address
Full Postal Code
Telephone Number
Email Address
Age at time of data collection

2.0 **Will you be collecting - at any time of the study, including recruitment of participants - any of the following** *(check all that apply)*:
Other Date (eg Date of Service)

3.0 * **If you are collecting any of the above, provide a comprehensive rationale to explain why it is necessary to collect this information:**
The contact information is necessary to be able to contact the patients if when analyzing the data we find reasons to refer the patients for medical care or investigation.
Personal information will also be used to query the clinic database and retrieve radiograph information and questionnaire information and link it with the rest of the study data. This linkage allows avoiding having to request that additional radiographs or questionnaires be completed.

4.0 **If identifying information will be removed at some point, when and how will this be done?**
During the patient visit. We will assign a code which will appear in all data collection forms and in the images saved. The code will be used to match the different pieces of data to the same patient. We will keep coded identifying information in a separate file for the PI in order to be able to contact patients who would potentially need a referral.

5.0 * **Specify what identifiable information will be RETAINED once data collection is complete, and explain why retention is necessary. Include the retention of master lists that link participant identifiers with de-identified data:**
All the information above will be retained but de-linked from the study data until all results have been analyzed and published in case findings suggest a referral of the patient is needed. After that time we will destroy the file containing the personal information.

6.0 **If applicable, describe your plans to link the data in this study with data associated with other studies (e.g within a data repository) or with data belonging to another organization:**
Personal identifiers above will be used to query the data from the Specialized scoliosis database from the respective sites.
Once extracted and copied to our study database, the date will be de-linked.

5.3 Data Confidentiality and Privacy

1.0 * **How will confidentiality of the data be maintained? Describe how the identity of participants will be protected both during and after research.**
Study document in locked filing cabinet, computer files on a password protected computer .

2.0 **How will the principal investigator ensure that all study personnel are aware of their responsibilities concerning participants' privacy and the confidentiality of their information?**
Basic CITI course in the Protection of Human Research Subject Nov. 2007 Paul Braunschweiger Ph.D., U. of Miami, online CITI Course Coordinator (Eric Parent)

FGSR online ethics course, section on ethics of REHAB 600 and 601 for Elise Watkins and Sanja Schreiber and UofA graduated Students.

Doug Hill is a member of the HREB panel B

Any other members of the personnel will sing the privacy and confidentiality agreement from their respective sites.
(EG AHS form for Edmonton and Calgary).

3.0 **External Data Access**

* **3.1 Will identifiable data be transferred or made available to persons or agencies outside the research team?**
○ Yes ● No

5.4 Data Storage, Retention, and Disposal

1.0 * **Describe how research data will be stored, e.g. digital files, hard copies, audio recordings, other. Specify the physical location and how it will be secured to protect confidentiality and privacy.** *(For example, study documents must be kept in a locked filing cabinet and computer files are encrypted, etc. Write N/A if not applicable to your research)*
Data will be in password protected files and in locked cabinets in Dr. Parent's lab.

2.0 * **University policy requires that you keep your data for a minimum of 5 years following completion of the study but there is no limit on data retention. Specify any plans for future use of the data. If the data will become part of a data repository or if this study involves the creation of a research database or registry for future research use, please provide details.** *(Write N/A if not applicable to your research)*
At this time we do not expect the data to be part of a repository for future use.

3.0 **If you plan to destroy your data, describe when and how this will be done? Indicate your plans for the destruction of the identifiers at the earliest opportunity consistent with the conduct of the research and/or clinical needs:**
Once analyses are completed and results published we will destroy the personal identifier files.

Documentation

Add documents in this section according to the headers. Use Item 11.0 "Other Documents" for any material not specifically mentioned below.

Sample templates are available by clicking HERE.

1.0 **Recruitment Materials:**

Document Name	Version	Date	Description
Multicenter SETS study info poster(0.03)	0.03	10/1/2013 9:16 AM	

2.0 Letter of Initial Contact:

Document Name	Version	Date	Description
letter of initial contact revised(0.01)	0.01	9/14/2010 10:59 PM	

3.0 Informed Consent / Information Document(s):

3.1 What is the reading level of the Informed Consent Form(s):
Assent form reading ease is 5.8; Parental consent form is 7.7: for Information Sheet is 8.8.

3.2 Informed Consent Form(s)/Information Document(s):

Document Name	Version	Date	Description
Parental consent revised(0.02)	0.02	10/2/2013 11:37 AM	
INFORMATION SHEET Nov 8th clean.doc(0.08)	0.08	11/8/2013 10:12 AM	
Phone script for COVID19 consent addendum discussion(0.01)	0.01	9/10/2020 2:18 PM	
CONSENT ADDENDUM FOR COVID(0.01)	0.01	9/10/2020 2:19 PM	

4.0 Assent Forms:

Document Name	Version	Date	Description
Assent Oct 1 2013(0.04)	0.04	10/2/2013 12:00 PM	

5.0 Questionnaires, Cover Letters, Surveys, Tests, Interview Scripts, etc.:

Document Name	Version	Date	Description

There are no items to display

There are no items to display

6.0 Protocol/Research Proposal:

Document Name	Version	Date	Description
SRS Small Exploratory Grant Application(0.01)	0.01	8/4/2010 2:09 PM	
Glenrose Rehabilitation Hospital Research Grant(0.01)	0.01	8/4/2010 2:12 PM	
SICKKIDS PROPOSAL MULTICENTER SETS (FUNDED)(0.01)	0.01	10/1/2013 11:11 AM	

7.0 Investigator Brochures/Product Monographs:

Document Name	Version	Date	Description

There are no items to display

8.0 Health Canada No Objection Letter ***(NOL)*****:**

Document Name	Version	Date	Description

There are no items to display

9.0 Confidentiality Agreement:

Document Name	Version	Date	Description

There are no items to display

10.0 Conflict of Interest:

Document Name	Version	Date	Description

There are no items to display

11.0 Other Documents:
For example, Study Budget, Course Outline, or other documents not mentioned above

Document Name	Version	Date	Description
Letter to primary care physicians (0.02)	0.02	10/1/2013 11:19 AM	

ID: Pro00043397 **Pro00043397** Final Page
Status: Approved

Final Page

You have reached the end of the ethics application.
Click 'Continue' or 'Exit' below.

To submit for ethics review, click "SUBMIT for REVIEW" on the left side of the screen.

NOTE: Only the Principal Investigator can submit an application in Pre-submission (ie: the first time it is submitted).

ID: Pro00043397 **Pro00043397** Add/Edit Funding Info without Manual Entry
Status: Approved

If you are trying to add a RES number in the ARISE application and you cannot find it on the drop down menu, please check the following:

1. *Check that the named investigators on your application match the people named on the RES account. RES numbers associated with anyone named as PI or Co-I on an ethics application will show up in the drop down box in Section 1.3 or in 6.0 of the Change Funding Activity. Please note that unless someone is named on the ethics application in either the PI or Co-I fields, their RES number(s) will NOT display in the drop down box of that application.*
2. *Check that the RES number you are trying to add has been activated by RSO (check unit name with RSO) and that 24 hours have elapsed since it was activated to allow time for system updates.*

If neither of the above items are the source of the issue, please contact reoffice@ualberta.ca.

Enter your Peoplesoft Project ID (aka RES#) to link this ethics application to the project record in PeopleSoft.

PeopleSoft Project ID:

Other Relevant Information:

ID: Pro00043397 **Pro00043397** Add/Edit Funding Info without Manual Entry
Status: Approved

If you are trying to add a RES number in the ARISE application and you cannot find it on the drop down menu, please check the following:

1. *Check that the named investigators on your application match the people named on the RES account. RES numbers associated with anyone named as PI or Co-I on an ethics application will show up in the drop down box in Section 1.3 or in 6.0 of the Change Funding Activity. Please note that unless someone is named on the ethics application in either the PI or Co-I fields, their RES number(s) will NOT display in the drop down box of that application.*
2. *Check that the RES number you are trying to add has been activated by RSO (check unit name with RSO) and that 24 hours have elapsed since it was activated to allow time for system updates.*

If neither of the above items are the source of the issue, please contact reoffice@ualberta.ca.

Enter your Peoplesoft Project ID (aka RES#) to link this ethics application to the project record in PeopleSoft.

PeopleSoft Project ID:

Other Relevant Information:

ID: Pro00043397 **Pro00043397**

Status: Approved Add/Edit Funding Info without Manual Entry

If you are trying to add a RES number in the ARISE application and you cannot find it on the drop down menu, please check the following:

1. *Check that the named investigators on your application match the people named on the RES account. RES numbers associated with anyone named as PI or Co-I on an ethics application will show up in the drop down box in Section 1.3 or in 6.0 of the Change Funding Activity. Please note that unless someone is named on the ethics application in either the PI or Co-I fields, their RES number(s) will NOT display in the drop down box of that application.*
2. *Check that the RES number you are trying to add has been activated by RSO (check unit name with RSO) and that 24 hours have elapsed since it was activated to allow time for system updates.*

If neither of the above items are the source of the issue, please contact reoffice@ualberta.ca.

Enter your Peoplesoft Project ID (aka RES#) to link this ethics application to the project record in PeopleSoft.

PeopleSoft Project ID:

Other Relevant Information:
SickKids New Investigator Grant NI14-018R
