# Supplementary material for: Effect of adding Schroth physiotherapeutic scoliosis specific exercises to standard care in adolescents with idiopathic scoliosis on posture assessed using surface topography: A secondary analysis of a Randomized Controlled Trial (RCT)
Source: PLoS One. 2024 Apr 30;19(4):e0302577. doi: 10.1371/journal.pone.0302577 (PMC11060560; doi:10.1371/journal.pone.0302577)
Supplement: S3 File — (DOCX) [file pone.0302577.s003.docx]

Linear mixed effects model coefficients, standard error, and significance values for estimating RMS and MaxDev in the analysis between groups. Model outputs for intention to treat and per protocol analysis are reported. Covariables of the mixed effects model are the following: Age, height, weight, if participants are braced, and their curve classification.

| **Linear Mixed Effects Model Outputs for Estimating RMS** | | | | | | |
| --- | --- | --- | --- | --- | --- | --- |
|  | **Intention to Treat** | | | **Per Protocol** | | |
| **Term** | **Estimate** | **Standard Error** | **p-value** | **Estimate** | **Standard Error** | **p-value** |
| **Intercept** | -1.26 | 6.62 | 0.85 | 2.19 | 8.63 | 0.80 |
| **[Group=0]*[Time=3]** | -1.25 | 0.71 | 0.08 | -1.66 | 0.98 | 0.09 |
| **[Group=0]*[Time=2]** | -0.94 | 0.75 | 0.21 | -1.212 | 0.97 | 0.21 |
| **[Group=0]*[Time=1]** | -1.33 | 0.73 | 0.07 | -1.53 | 0.98 | 0.12 |
| **[Group=1]*[Time=3]** | -1.17 | 0.33 | **0.001** | -1.63 | 0.44 | **0.0004** |
| **[Group=1]*[Time=2]** | -0.62 | 0.33 | 0.06 | -0.72 | 0.42 | 0.09 |
| **Age** | 1.08 | 0.28 | **0.0001** | 1.13 | 0.35 | **0.001** |
| **Height** | -0.02 | 0.06 | 0.79 | -0.04 | 0.08 | 0.57 |
| **Weight** | 0.00 | 0.05 | 0.97 | 0.003 | 0.08 | 0.97 |
| **Braced** | 1.26 | 0.71 | 0.08 | 1.38 | 0.95 | 0.15 |
| **3cp Curve** | 3.01 | 1.18 | **0.01** | 2.34 | 1.59 | 0.15 |
| **4c Curve** | -0.73 | 1.37 | 0.59 | -1.99 | 1.78 | 0.26 |
| **4cp Curve** | 1.11 | 1.16 | 0.34 | 1.49 | 1.51 | 0.32 |
| **Linear Mixed Effects Model Outputs for Estimating MaxDev** | | | | | | |
|  | **Intention to Treat** | | | **Per Protocol** | | |
| **Term** | **Estimate** | **Standard Error** | **p-value** | **Estimate** | **Standard Error** | **p-value** |
| **Intercept** | -5.17 | 11.11 | 0.64 | 0.80 | 14.69 | 0.96 |
| **[Group=0]*[Time=3]** | -2.58 | 1.19 | **0.03** | -4.17 | 1.65 | **0.01** |
| **[Group=0]*[Time=2]** | -1.64 | 1.22 | 0.18 | -2.73 | 1.64 | 0.10 |
| **[Group=0]*[Time=1]** | -2.47 | 1.20 | **0.04** | -3.14 | 1.65 | 0.06 |
| **[Group=1]*[Time=3]** | -1.93 | 0.52 | **0.0002** | -2.93 | 0.72 | **0.0001** |
| **[Group=1]*[Time=2]** | -1.20 | 0.51 | **0.02** | -1.71 | 0.68 | **0.013** |
| **Age** | 1.74 | 0.46 | **0.0002** | 1.83 | 0.59 | **0.002** |
| **Height** | -0.01 | 0.10 | 0.90 | -0.07 | 0.13 | 0.60 |
| **Weight** | 0.00 | 0.09 | 0.99 | 0.04 | 0.13 | 0.79 |
| **Braced** | 0.82 | 1.19 | 0.49 | 1.32 | 1.62 | 0.42 |
| **3cp Curve** | 3.86 | 1.95 | **0.05** | 3.17 | 2.72 | 0.25 |
| **4c Curve** | -2.20 | 2.26 | 0.33 | -3.47 | 3.03 | 0.25 |
| **4cp Curve** | 1.24 | 1.91 | 0.52 | 2.51 | 2.57 | 0.33 |

Post hoc comparison of estimated marginal mean from fitted linear mixed effects models of RMS and MaxDev outcomes. Comparisons for intention to treat and per protocol analyses reported. Control group coded a 0, Schroth group coded as 1. Baseline, 3 months, and 6 months variables were coded as Time =1, Time=2, and Time=3, respectively.

| **RMS Estimated Mean Difference Comparison** | | | | |
| --- | --- | --- | --- | --- |
|  | **Intention to treat** | | **Per Protocol** | |
| **Comparison** | **Mean Estimate Difference** | **p-value** | **Mean Estimate Difference** | **p-value** |
| Group=0,Time=3 - Group=0,Time=2 | -0.31 | 0.44 | -0.45 | 1.00 |
| Group=0,Time=3 - Group=0,Time=1 | 0.08 | 0.81 | -0.13 | 1.00 |
| Group=0,Time=3 - Group=1,Time=3 | 0.19 | 0.80 | -0.04 | 1.00 |
| Group=0,Time=3 - Group=1,Time=2 | -0.37 | 0.62 | -0.95 | 1.00 |
| Group=0,Time=3 - Group=1,Time=1 | -0.99 | 0.18 | -1.66 | 0.76 |
| Group=0,Time=2 - Group=0,Time=1 | 0.39 | 0.30 | 0.32 | 1.00 |
| Group=0,Time=2 - Group=1,Time=3 | 0.50 | 0.53 | 0.42 | 1.00 |
| Group=0,Time=2 - Group=1,Time=2 | -0.06 | 0.94 | -0.49 | 1.00 |
| Group=0,Time=2 - Group=1,Time=1 | -0.68 | 0.38 | -1.21 | 0.97 |
| Group=0,Time=1 - Group=1,Time=3 | 0.10 | 0.89 | 0.09 | 1.00 |
| Group=0,Time=1 - Group=1,Time=2 | -0.45 | 0.55 | -0.82 | 1.00 |
| Group=0,Time=1 - Group=1,Time=1 | -1.07 | 0.16 | -1.53 | 0.85 |
| Group=1,Time=3 - Group=1,Time=2 | -0.55 | 0.12 | -0.91 | 0.40 |
| Group=1,Time=3 - Group=1,Time=1 | -1.17 | **0.001** | -1.63 | **0.01** |
| Group=1,Time=2 - Group=1,Time=1 | -0.62 | 0.06 | -0.72 | 0.77 |
| **MaxDev Estimated Mean Difference Comparison** | | | | |
|  | **Intention to Treat** |  | **Per Protocol** |  |
| **Comparison** | **Mean Estimate Difference** | **p-value** | **Mean Estimate Difference** | **p-value** |
| Group=0,Time=3 - Group=0,Time=2 | -0.95 | 0.16 | -1.44 | 0.48 |
| Group=0,Time=3 - Group=0,Time=1 | -0.11 | 0.85 | -1.03 | 0.94 |
| Group=0,Time=3 - Group=1,Time=3 | -0.30 | 0.81 | -1.24 | 1.00 |
| Group=0,Time=3 - Group=1,Time=2 | -1.03 | 0.40 | -2.46 | 0.89 |
| Group=0,Time=3 - Group=1,Time=1 | -2.23 | 0.07 | -4.17 | 0.17 |
| Group=0,Time=2 - Group=0,Time=1 | 0.84 | 0.15 | 0.41 | 1.00 |
| Group=0,Time=2 - Group=1,Time=3 | 0.65 | 0.60 | 0.20 | 1.00 |
| Group=0,Time=2 - Group=1,Time=2 | -0.08 | 0.95 | -1.02 | 1.00 |
| Group=0,Time=2 - Group=1,Time=1 | -1.28 | 0.30 | -2.73 | 0.79 |
| Group=0,Time=1 - Group=1,Time=3 | -0.19 | 0.88 | -0.21 | 1.00 |
| Group=0,Time=1 - Group=1,Time=2 | -0.92 | 0.45 | -1.43 | 1.00 |
| Group=0,Time=1 - Group=1,Time=1 | -2.12 | 0.09 | -3.14 | 0.60 |
| Group=1,Time=3 - Group=1,Time=2 | -0.73 | 0.18 | -1.22 | 0.68 |
| Group=1,Time=3 - Group=1,Time=1 | -1.93 | **0.0002** | -2.93 | **0.001** |
| Group=1,Time=2 - Group=1,Time=1 | -1.20 | 0.02 | -1.71 | 0.18 |

Linear mixed effects model coefficients, standard error, and significance values for estimating RMS and MaxDev in the analysis within controls when receiving delayed Schroth exercises in that last 6 months of the 1-year follow-up. Model outputs for intention to treat and per protocol analysis are reported. Covariables of the mixed effects model are the following: Age, height, weight, if participants are braced, and their curve classification.

| **Mixed Effect Model Outputs for Estimating RMS** | | | | | | |
| --- | --- | --- | --- | --- | --- | --- |
|  | **Intention to Treat** | | | **Per Protocol** | | |
| **Term** | **Estimate** | **Standard Error** | **p-value** | **Estimate** | **Standard Error** | **p-value** |
| **Intercept** | 4.44 | 8.78 | 0.61 | -4.92 | 14.26 | 0.73 |
| **Time=3** | 0.58 | 0.49 | 0.25 | -0.77 | 0.57 | 0.19 |
| **Time=2** | 0.50 | 0.55 | 0.38 | -0.52 | 0.52 | 0.32 |
| **Age** | -0.06 | 0.08 | 0.41 | 1.35 | 0.54 | **0.01** |
| **Height** | 0.08 | 0.07 | 0.30 | -0.06 | 0.13 | 0.64 |
| **Weight** | 0.64 | 0.94 | 0.49 | 0.12 | 0.10 | 0.26 |
| **Braced** | 2.20 | 1.74 | 0.21 | 1.23 | 1.25 | 0.33 |
| **3cp Curve** | -0.89 | 2.09 | 0.67 | 1.28 | 2.56 | 0.62 |
| **4c Curve** | 1.29 | 1.70 | 0.45 | 0.49 | 3.28 | 0.88 |
| **4cp Curve** | 4.44 | 8.78 | 0.61 | 1.18 | 2.49 | 0.64 |
| **Mixed Effect Model Outputs for Estimating MaxDev** | | | | | | |
|  | **Intention to Treat** | | | **Per Protocol** | | |
| **Term** | **Estimate** | **Standard Error** | **p-value** | **Estimate** | **Standard Error** | **p-value** |
| **Intercept** | 0.95 | 15.67 | 0.95 | -10.97 | 26.16 | 0.68 |
| **Time=3** | 1.35 | 0.70 | 0.06 | -0.76 | 0.93 | 0.42 |
| **Time=2** | 0.47 | 0.71 | 0.51 | -1.17 | 0.83 | 0.16 |
| **Age** | -0.07 | 0.14 | 0.62 | 2.50 | 0.99 | **0.01** |
| **Height** | 0.07 | 0.13 | 0.56 | -0.07 | 0.23 | 0.76 |
| **Weight** | -0.13 | 1.70 | 0.94 | 0.11 | 0.19 | 0.56 |
| **Braced** | 2.04 | 3.23 | 0.53 | 0.85 | 2.30 | 0.71 |
| **3cp Curve** | -3.18 | 3.57 | 0.37 | -2.72 | 4.70 | 0.57 |
| **4c Curve** | 0.60 | 3.07 | 0.84 | -3.13 | 6.02 | 0.61 |
| **4cp Curve** | 0.95 | 15.67 | 0.95 | -1.77 | 4.57 | 0.70 |
